# PCNA regulates primary metabolism by scaffolding metabolic enzymes

**DOI:** 10.1038/s41388-022-02579-1

**Published:** 2022-12-23

**Authors:** Lisa M. Røst, Synnøve B. Ræder, Camilla Olaisen, Caroline K. Søgaard, Animesh Sharma, Per Bruheim, Marit Otterlei

**Affiliations:** 1 Department of Biotechnology and Food Science, Faculty of Natural Sciences, NTNU Norwegian University of Science and Technology, NO-7491 Trondheim, Norway; 2 Department of Clinical and Molecular Medicine, Faculty of Medicine and Health Sciences, NTNU Norwegian University of Science and Technology, NO-7491 Trondheim, Norway; 3 Proteomics and Modomics Experimental Core Facility (PROMEC), NTNU, NO-7491 Trondheim, Norway; 4 Clinic of Surgery, St. Olavs Hospital, Trondheim University Hospital, N-7006 Trondheim, Norway; 5 APIM Therapeutics A/S, Trondheim, Norway

**Keywords:** Growth factor signalling, Proteomics, Metabolism, Molecular biology

## Abstract

The essential roles of proliferating cell nuclear antigen (PCNA) as a scaffold protein in DNA replication and repair are well established, while its cytosolic roles are less explored. Two metabolic enzymes, alpha-enolase (ENO1) and 6-phosphogluconate dehydrogenase (6PGD), both contain PCNA interacting motifs. Mutation of the PCNA interacting motif APIM in ENO1 (F423A) impaired its binding to PCNA and resulted in reduced cellular levels of ENO1 protein, reduced growth rate, reduced glucose consumption, and reduced activation of AKT. Metabolome and signalome analysis reveal large consequences of impairing the direct interaction between PCNA and ENO1. Metabolites above ENO1 in glycolysis accumulated while lower glycolytic and TCA cycle metabolite pools decreased in the APIM-mutated cells; however, their overall energetic status were similar to parental cells. Treating haematological cancer cells or activated primary monocytes with a PCNA targeting peptide drug containing APIM (ATX-101) also lead to a metabolic shift characterized by reduced glycolytic rate. In addition, we show that ATX-101 treatments reduced the ENO1 - PCNA interaction, the ENO1, GAPDH and 6PGD protein levels, as well as the 6PGD activity. Here we report for the first time that PCNA acts as a scaffold for metabolic enzymes, and thereby act as a direct regulator of primary metabolism.

## Introduction

PCNA is a member of the essential and highly conserved DNA sliding clamp family. PCNA interacts with proteins carrying either of the two conserved PCNA interacting motifs; the PCNA interacting peptide (PIP) – box [1] or the AlkB homolog 2 PCNA interacting motif (APIM) [2]. The two motifs share binding site on PCNA [3, 4]. Multi-layered regulatory mechanisms, including affinity-driven competition and posttranslational modifications (PTMs) of PCNA, control which of the up to 600 proteins [5] that may interact with PCNA through these two motifs at any given time. For example, while replication is strictly dependent upon high affinity canonical PIP-box - PCNA interactions, the affinity of APIM is increased after stress-induced PTMs such as poly-ubiquitination of PCNA [2, 6, 7]. Increased affinity of APIM during stress is in accordance with the ability of cell-penetrating peptides containing APIM (APIM-peptides) to increase the efficacy of both genotoxic drugs and drugs targeting microtubules or kinases without inhibiting normal replication [3, 5, 8–12]. An experimental APIM-peptide drug, ATX-101, currently in Phase II clinical trials (ATX-101, NCT05116683 and NCT04814875), has shown a favourable toxicity profile in Phase I and disease stabilization in late stage solid tumor patients without induction of myelosuppression [13]. This supports that the APIM-peptide is targeting PCNA’s role in stress without affecting replication.

Although PCNA is mostly known for its canonical roles in DNA replication and repair, increasing evidence supports multiple cytosolic roles: **I**) Cytosolic PCNA regulates apoptosis by binding to procaspases in mature neutrophils [14], multiple myeloma (MM) cells [3], and neuronal cells [15]; **II**) Cytosolic PCNA in cancer cells inhibits activation of natural killer cells, thereby helping cancer cells to evade the immune system [16]; **III**) Multiple proteins involved in PI3K/AKT and MAPK signalling were found in PCNA complexes [10], and targeting PCNA with an APIM-peptide was linked to downregulation of EGFR and PI3K/AKT pathways and inhibition of cytokine production from activated monocytes [9, 10]; **IV**) The role of PCNA as a scaffold protein in MAPK signalling is functionally conserved between mammalians and yeast [5]; **V**) Cytosolic PCNA participates in reactive oxygen species regulation via interaction with a subunit of the NADPH oxidase complex [17]; **VI**) Nuclear export of PCNA correlates with increased Warburg-effect in acute myeloid leukaemia (AML) cells [18]; and seven glycolytic enzymes are reported to be in complex with PCNA. These are aldolase, triosephosphate isomerase, glyceraldehyd-3-phosphate dehydrogenase (GAPDH), phosphoglycerate kinase, phosphoglycerate mutase, alpha-enolase (ENO1) and pyruvate kinase [19], catalysing step 4-10 of glycolysis. One of these enzymes, ENO1, contains a putative APIM. ENO1 catalyses the conversion of 2-phosphoglycerate (2PG) to phosphoenolpyruvate (PEP). In addition, 6-phosphogluconate dehydrogenase (6PGD), catalysing the third step of the pentose phosphate pathway (PPP), contains a putative PIP-box, and is thus another potential PCNA-interacting metabolic enzyme [2, 5]. Complexes containing PCNA and metabolic enzymes could possibly act as “metabolons” in which PCNA serves a scaffold function. The term “metabolon” was introduced almost forty years ago, describing temporary structure-function complexes of metabolic enzymes that facilitate metabolite channelling [20], and has lately been exemplified by identification of the “purinosome”, channelling *de novo* purine synthesis [21].

Here we show that ENO1 directly interacts with PCNA via its APIM, and that mutation of this motif impairs the PCNA interactions and reduce the cellular levels of ENO1. Furthermore, these cells grew slower, consumed less glucose, had altered central carbon metabolite pools, and reduced activation of AKT compared to the parental cells. Targeting stress-mediated protein - PCNA interactions using ATX-101 reduced the levels of glycolytic and PPP intermediates and the protein levels of ENO1, GAPDH and 6PGD. Altogether, these results support that PCNA can serve a role as a scaffold protein in glycolytic metabolons.

## Material and Methods

Cell densities, treatment concentrations, number of replicate cell cultures and repeated experiments, and sampling time points listed for all individual assays and cell lines/types are given in Supplementary Table S1.

### Peptides and stressors

APIM-peptide; ATX-101 (Ac-MDRWLVKWKKKRKIRRRRRRRRRRR), mutAPIM-peptide; ATX-A (Ac-MDRALVKWKKKRKIRRRRRRRRRRR) [3], and R11 (Ac-RRRRRRRRRRR) [10] (Innovagen, Lund, Sweden) were used. Monocytes were stimulated with LPS (Sigma-Aldrich, Saint-Louis, Missouri, USA) for 4 h, and cell lines were treated with cisplatin (Hospira, Lake Forest, Illinois, USA).

### Cell lines and assay conditions

Cell densities were optimized for each individual assay and corresponding sampling time points, and peptide doses were adjusted to cell density to obtain the same number of annexin-positive cells in all experiments. These data are listed in Supplementary Table S1, also listing number of independent cultures analysed (n), number of repeated experiments and time of harvest.

JJN3 [3] and RPMI 8226 (ATCC, Manassas, Virginia, USA, CCL-155) (MM), HL60 (ATCC CCL-240) and NB4 (Kind gift from professor Stein Døskeland, University of Bergen, Norway) (AML), and DU145 (ATCC HBT-81, prostate cancer) cells were cultured in RPMI 1640 (Sigma-Aldrich), MC/CAR (ATTC CRL-8083) (MM) were cultured in IMDM (Sigma-Aldrich or Gibco, Thermo Fisher Scientific, Waltham, Massachusetts, USA), and HEK293 (ATCC CRL-1573, embryonic kidney) were cultured in DMEM high glucose (Sigma-Aldrich,), all supplemented with 2 mM glutamine (Biochrom, Berlin, Germany), 100 µg/mL gentamicin (Sigma-Aldrich or Gibco), 2.5 µg/mL amphotericin (Sigma-Aldrich), and fetal bovine serum (FBS)(Sigma-Aldrich); 10% in DMEM high glucose and RPMI 1640, and 20% in IMDM. Cells were maintained at 37 °C in a humidified atmosphere of 5% CO_2_.

### Primary monocytes

Peripheral blood monocytes were isolated and cultured from three A + /− buffy coats (Blood Bank, St. Olav’s University Hospital, Norway) as described [10].

### Mutated cell lines

HAP1 a near haploid cell line isolated from a chronic myeloid leukaemia patient was selected for the site-specific mutation because it is easier to make mutations in a haploid than in a diploid cell. CRISPR/Cas9-edited HAP1 ENO1 F423A cell lines were ordered from Horizon (Cambridge, UK), and two individual clones (called M1 and M2) and the parental cell line (WT) were used here. The mutation was verified by targeted sequencing (non-allele specific PCR). The cell lines were cultured in IMDM (ThermoFisher Scientific, Waltham, MA, USA) supplemented with 10 % FBS, 2.5 µg/ml Fungizone® Amphotericin B (Gibco), 1x MEM non-essential amino acids Solutions (Gibco, or Sigma-Aldrich) and antibiotic mixture containing 100 µg/ml penicillin and 100 µg/ml streptomycin (Gibco). The cells were maintained at 37 °C in a humidified atmosphere of 5% CO_2_.

### Viability assay

PrestoBlue viability assay was performed according to the manufacturer’s guidelines. Briefly, cells were seeded in 96 well plates at a concentration of 3000 cells/well. Further, cells were treated with PrestoBlue™ Cell Viability Reagent (1:10 dilution, Invitrogen, Thermo Fischer Scientific) for three consecutive days cells. Fluorescence was read after 1.5 h at (excitation 544, emission 590 nm) on a plate reader (FLUOstar Omega microplate reader, BMG LABTECH).

### Cell extracts

HAP1 cells were seeded out (100 000 cells/mL) the day before treatment with ATX-101. ATX-101 was added to existing media to final a concentration of 20 μM 1 to 4 times with 4 h interval (0, 4, 8, 12 h), assuming that previously added ATX-101 was broken down and/or taken up by the cells. HAP1 cells were harvested 24 h after the first treatment, and at harvest the viability of treated cells relative to untreated cells was reduced by 0%, 5%, 12% and 51% respectively for cells treated 1, 2, 3, and 4 times with ATX-101. JJN3, MC/CAR, NB4, primary monocytes and HAP1 cells were harvested, by scraping if adherent, by centrifugation (5000 x g, 5 min, 4 °C) and lysed in M-PER buffer (3x packed cell volume, Thermo Fischer Scientific) with Halt protease and phosphatase inhibitor cocktail (1x, Thermo Fischer Scientific), DTT (1 mM) and omnicleave (1 μL, Lucigen, OC7850K), incubated 1.5 h, on ice with vortexing every 30 min, before the supernatant (the cell extract) was collected.

Cytosolic cell extracts from HAP1 cells were made by resuspending cell pellets (1:1 volume) with lysis buffer (40 mM HEPES, 120 mM KCl, 2 mM EGTA, 10 mM β-glycerophosphate, 0.4% NP-40 (Thermo Fisher Scientific) and protease inhibitor cocktail) followed by 30 min incubation on 4 °C with rotation. The cytosolic fraction was obtained by centrifugating the lysate 1000 x g for 5 min to pellet the nuclei, followed by centrifugation of the supernatant 10,000 x g for 10 min.

### Immunoprecipitation (IP)

Dynabeads Protein A (Invitrogen) were washed with conjugation buffer (20 mM NaP and 0.15 M NaCl pH 7.9) and incubated in conjugation buffer with 3.3 ug antibody/mg beads of α-ENO1 or α-PCNA (Abcam Cambridge, UK, ab227978 and PC10, Santa Cruz Biotechnology, Dallas, Texas, USA, Sc-56 respectively) for 1.5 h. The conjugated beads were crosslinked with BS^3^ (bis(sulfosuccinimidyl)suberate, Thermo Fisher Scientific) according to the manufacturer’s manual. Briefly, the antibody conjugated beads were washed twice with conjugation buffer before incubation with 5 mM BS^3^ (in 5x volume of beads) for 30 min. The reaction was quenched adding Tris-HCl pH 7.5 (20 mM, final concentration). The beads were washed three times with IP buffer I (20 mM HEPES, 1.5 mM MgCl_2_, 200 mM KCl, 0.2 mM EGTA, 20% (v/v) glycerol and 0.5% NP-40) and finally resuspended in IP buffer II (as IP buffer I except 10% (v/v) glycerol and without NP-40). Each IP reaction, containing 2 mg cytosolic cell extract, 3 mg beads (conjugated with ~10 μg antibody) and IP buffer II up to a total of 500 μl for each sample, was incubated at 4 °C overnight on rotation. Further, the IP reactions were washed once with IP buffer II and three times with washing buffer (10 mM Tris-HCl pH 7.5 and 600 mM NaCl) before elution by incubation on 70 °C for 15 min in 4x LDS and 1 mM DTT. The samples were run on gel and blotted and developed as described.

### Western blot

Cell extracts (150 μg protein for JJN3 cells and 50 μg from HAP1 cells) were added DTT (0.1 M) and LDS loading buffer (4x, Life Technologies, Carlsbad, California, USA) and incubated to reverse cross-links (10 min, 70 °C). Protein from IP or cell extracts were separated by electrophoresis (4–12% Bis-Tris gels, NuPAGE, Invitrogen) and blotted to polyvinylidene fluoride membranes (0.2 μM, Immobilon, Merch Millipore, Burlington, Massachusetts, USA). The membranes were blocked in 5% drymilk in TBS (1 h) or Intercept (PBS) blocking buffer (LI-COR Biosciences, Lincoln, Nebraska, USA) before incubation with primary antibody against ENO1 (abcam, ab227978 or ab155102, 1:1000), 6PGD (Cell Signaling Technology, Danvers, Massachusetts, USA, #13389, 1:500 or Santa Cruz Biotechnology, sc-398977, 1:250), GAPDH (Abcam, ab8245, 1:1000), PCNA (Santa Cruz Biotechnology, PC10, Sc-56, 1:2000), AKT (Cell Signaling Technology, #4691, 1:1000), Phospho-AKT (Ser473, Cell Signaling Technologies, #4060, 1:2000) or β-actin (Abcam, ab8226, 1:2000) and H3 (Abcam, ab1791, 1:2000) used as loading controls. The fluorescently labelled secondary antibodies IRdye 680RD goat α-mouse (LI-COR, #925-68070, 1:20 000) and IRdye 800CW goat α-rabbit (LI-COR, #926-32211, 1:20 000) were used for protein detection. All antibodies were diluted in blocking buffer with 0.1% Tween 20 (Sigma Aldrich), overnight (primary antibodies) or 1 h (secondary antibodies). Proteins were visualized in the Odyssey infrared imaging system (LI-COR Biosciences and quantified in Odyssey Image Studio (V2.0). Protein levels were normalized against β-actin, or against PCNA/ENO1 for the IPs.

### ENO1 APIM-YFP constructs

Oligos containing ENO1 APIM (KFAGR) and a mutated version (KAAGR) were designed (Sigma) to fit in pEYFP plasmid between restriction sites XhoI and EcoRI. The oligos were annealed and ligated into XhoI/EcoRI cut pEYFP plasmid and sequenced to validate the correct constructs (pEYFP-KFAGR or pEYFP-KAAGR). Oligo-pair 1 designed for the KFAGR olio (APIM sequence underlined) and oligo-pair 2 designed for the KAAGR oligo (mutated APIM sequence underlined): 1) 5’-Phos-TCGAGATGGCTAAGTTTGCCGGCAGGAACTTCGG (forward) and 5’-Phos-AATTCCGAAGTTCCTGCCGGCAAA-CTTAGCCATC (reverse); 2) 5’-Phos-TCGAGATGGCTAAGGCTGCCGGCAGGAACTTCGG (forward) and 5’-Phos-AATTCCGAAGTTCCTGCCGGCAGCCTTAGCCATC (reverse).

### Confocal microscopy

Co-localization were examined by co-transfections of ENO1-APIM constructs (KFAGR or KAAGR) and CFP-PCNA [2] in HEK293T cells by using X-tremeGENE™ HP DNA Transfection Reagent (Roche, Mannheim, Germany) as described by the manufacturer. Live cells were visualized using Zeiss LSM 510 Meta laser confocal microscope (Carl Zeiss, Oberkochen, Germany) with ×63/1.4 oil immersion objective 24 h after transfection. CFP and YFP were excited at λ = 458 nm and λ = 514 nm and detected 470–500 nm and > 515 nm, respectively. For immunofluorescence (IF) of endogenous localization of ENO1 and PCNA, HAP1 cells (WT and mutant cell lines) were fixed (2% paraformaldehyde in PBS) for 10 min on ice and treated with methanol for 20 min (−20 °C) before incubated overnight at 4 °C with primary antibodies ENO1 (Abcam, ab155102) and PCNA (Santa Cruz Biotechnology, Sc-56) diluted 1:100 or 1:400 respectively in 2% FBS /PBS. Next, the cells were washed three times with FBS/PBS before and after incubation with secondary antibodies for 45 min (Life Technologies, Alexafluor 532 goat α-rabbit, Alexafluor 647 goat α-mouse, respectively, diluted 1:2000). For the proximity ligation assay (PLA) we used the same fixation procedure and the same primary antibodies as for IF, and next followed the instructions enclosed in the Naveniflex MR kit (Navinci, Sweden). The cells were visualized on Zeiss LSM 510 Meta laser confocal microscope using × 63/1.4 oil immersion objective and excited/ detected at λ = 514/ 530–600 nm and λ = 638 / > 650 nm, respectively. Treatment with ATX-101: HAP1 cells were seeded out (100,000 cells/mL) the day before treatment with ATX-101. ATX-101 was added to existing media to final a concentration of 20 μM 5 times with 1 h interval assuming that most of previously added ATX-101 was broken down and/or taken up by the cells. HAP1 cells were fixed 1–4 h after the last treatment and analysed by IF and PLA. At harvest, viable measured by the PrestoBlue viability assay was ~50%.

### 6PGD-assay

The 6PGD activity was measured using the 6-phospho gluconate dehydrogenase assay kit (Abcam, ab241016). In this assay, 6PGD catalyses the conversion of 6-phosphogluconate in a reaction generating NADPH that subsequently reduces a colourless probe to a coloured product that can be read at absorbance 460 nm. The assay was conducted as described by the supplier. Cells were harvested and lysed in 6PGD assay buffer and the supernatant collected. 6PGD activity was examined in lysates of differently treated cells and in lysates from untreated cells added the different treatments directly and immediately before measuring 6PGD activity. The lysates were added 6PGD developer and 6PGD substrate in a 96 wells plate and the absorbance measured (OD460 nm, 45 min, 37 °C) representing the amount of NADPH generated. The 6PGD activity was calculated as milliunits per mL (mU/mL) where one unit of 6PGD us the amount of enzyme that generates 1 μmol of NADPH per min at pH 8.0 at 37 °C.

### Measurements of extracellular metabolites

Extracellular glucose and lactate were measured in fresh and spent medium as described in [9].

### Targeted mass spectrometric metabolite profiling of intracellular metabolites

JJN3, RPMI 8226, MC/CAR, HL60 and NB4 cells were sampled as described for suspension cell lines [22]; by fast filtering at a maximum vacuum pressure of 250 mbar below the ambient pressure. HAP1, HEK293, and DU145 cells, and the primary monocytes were sampled as described for adherent cell lines [22], by mechanical detachment on a cold metal block. All sampled cells were immediately quenched in LN_2_, extracted, lyophilized and cleared of protein as described [22], and reconstituted in compatible solvents for downstream analysis. TCA cycle intermediates and phosphorylated metabolites were prepared for and quantified by capillary ion chromatography tandem mass spectrometry (capIC-MS/MS) as described [23], employing a Xevo triple quadrupole mass spectrometer (Waters, Milford, Maine, USA). The modifications described in [24] was employed for capIC-MS/MS analysis of all haematological cells. Amino acids in DU145 and HEK293 extracts were quantified by gas chromatography (GC)-MS/MS as described [25]. Amino acids in two out of three replicates of JJN3 extracts were derivatized and analysed as described [9]. Amino acids in all remaining haematological cell extracts were derivatized and analysed as described [22], so were lactate and pyruvate in MC/CAR and NB4 [22]. Figure 2C was created with Omix editor and modelling tool for metabolic network diagrams [26].

### Liquid chromatography (LC)-MS/MS analysis of pyridine nucleotide pools

Intracellular pyridine nucleotide pools were quantified as described [22].

### Multiplexed inhibitor bead (MIB)-assay

Cell extracts for the MIB-assay (kinase enrichment) were prepared as described above, and the MIB-assay were done as described [27].

### Statistical analysis

Statistical tests are given in the relevant figure legends. These include ANOVA, post hoc Turkey´s test, student´s T-test, and Wilcoxon Sign Rank test.

### MIB-assay data analysis

Proteins were quantified by processing MS data using MaxQuant v.1.6.17.0 [28]. Open workflow provided in FragPipe version 14 [28] was used to inspect the raw files to determine optimal search criteria. Namely, the following search parameters were used: enzyme specified as trypsin with a maximum of two missed cleavages allowed; acetylation of protein N-terminal, oxidation of methionine, deamidation of asparagine/glutamine, and phosphorylation of serine/threonine/tyrosine as dynamic post-translational modification. These were imported in MaxQuant which uses m/z and retention time (RT) values to align each run against each other sample with a minute window match-between-run function and 20 min overall sliding window using a clustering-based technique. These were further queried against the Human proteome including isoforms downloaded from Uniprot (https://www.uniprot.org/proteomes/UP000005640) in September 2019 along with MaxQuant’s internal contaminants database using Andromeda built into MaxQuant. Both protein and peptide identifications false discovery rate (FDR) was set to 1%, only unique peptides with high confidence were used for final protein group identification. Peak abundances were extracted by integrating the area under the peak curve. Each protein group abundance was normalized by the total abundance of all identified peptides for each run and protein by calculated median summing all unique and razor peptide-ion abundances for each protein using label-free quantification (LFQ) algorithm [29] minimum peptides ≥ 1. LFQ values for all samples were combined and log-transformed with base 2 and the transformed control values were subtracted. The resulting values reflecting the change relative to control for each condition were subjected to two-sided non-parametric Wilcoxon Sign Rank Test [30] as implemented in MATLAB R2015a (Math Works Inc, Natick, Massachusetts, USA) in order to check the consistency in directionality of the change, namely a negative sign reflecting decreased and positive sign reflecting increased expression of respective protein group. The choice of this non-parametric test avoids the assumption of a certain type of null distribution as in student’s t-test by working over the rank of the observation instead of observation value itself. Further, it also makes it robust to outliers and extreme variations noticed in observed values. Differentially expressed (DE) protein groups were identified at p 0.25. The Uniprot accession IDs of these DE were mapped to pathways (https://www.wikipathways.org/index.php/WikiPathways Pathways version wikipathways-20201010-gmt-Homo_sapiens.gmt (Carlson M (2019). org.Hs.eg.db: Genome wide annotation for Human. R package version 3.8.2.) using R (https://www.R-project.org/) libraries, org.Hs.eg.db and clusterProfiler (www.liebertpub.com/doi/10.1089/omi.2011.0118). Venn diagrams were built using the R package limma [31]. UniprotR package [32] and STRING analysis (https://string-db.org/) were used to map protein groups to their respective gene-ontology terms and pathways/biological processes. Online Ingenuity® Pathway Analysis™ (IPA) software (QIAGEN Inc., www.qiagenbioinformatics.com/products/ingenuitypathway-analysis) was used to combine with metabolomics data for annotation, visualization and integrated discovery of canonical pathways and other functional analysis.

### Quantification of extracellular metabolites

Extracellular glucose and lactate was quantified and normalized as described [9].

### Quantification of intracellular metabolites

Downstream data processing was performed in TargetLynx application manager of MassLynx 4.1 (Waters). Ion abundances were normalized to total ion abundance in DU145 and HEK293 extracts. Metabolite levels in HAP1, JJN3, RPMI 8226, MC/CAR, HL60, NB4, and primary monocyte extracts were absolutely quantified by interpolation from calibration curves prepared by serial dilutions of analytical grade standards (Sigma-Aldrich) calculated by least squares regression. Response factors of the analytical standards and biological extracts were corrected by the response factor of their corresponding U^13^C-labeled isotopologue spiked into the samples. Extract concentrations were then normalized to experimental cell density at the time of sampling, measured on a MoxiZ cell counter with type S cassettes (Orflo Technologies).

## Results

### Mutation of the PCNA interacting motif APIM reduces the stability of ENO1 and leads to phenotypic changes at growth, metabolite, and carbon flux levels

**Fig. 1:**
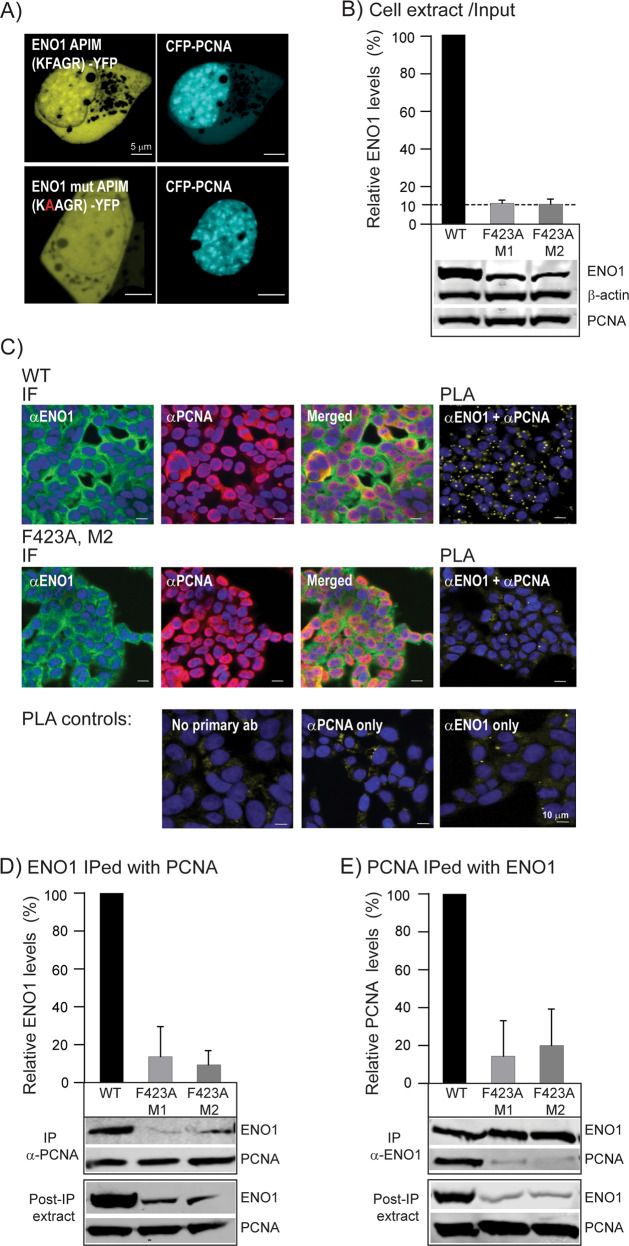
Mutation of APIM in ENO1 reduces binding to PCNA and ENO1 protein levels. **A** Images of HEK293 cells co-transfected with CFP-PCNA and ENO1 APIM (upper panel) and ENO1 mutated APIM (lower panel) fused to YFP. Scale bar: 5 µm. **B** Lower panel: representative western blot showing ENO1, PCNA and β-actin levels (input) in parental (WT) and ENO1 F423A (M1 and M2) HAP1 cytosolic cell extracts (50 µg). Upper panel: densitometric quantifications of ENO1 levels normalized to β-actin, presented relative to ENO1 levels in WT cells. Mean ± SD, *n* = 4. **C** Immunofluorescence (IF): confocal images of endogenous levels of ENO1 and PCNA in WT (upper panel) and F423A M2 cells (mid panel). α-ENO1 (green), α-PCNA (red), and DAPI (blue). Proximity Ligation Assay (PLA): α-ENO1 and α-PCNA (upper and mid panel), no primary antibodies (ab), only α-PCNA or only α-ENO1 (PLA controls, lower panel). Positive PLA signal (yellow spots). Scale bar: 10 µm. **D**, **E** Lower panels: representative western blots showing ENO1 IPed with PCNA (**D**) or PCNA IPed with ENO1 (**E**) from WT and F423A M1 and M2 extracts. WB of extracts post-IP shown, ~3% of the total IP solution added (input shown in **B**). Upper panels: densitometric quantifications of ENO1 normalized to PCNA levels (**D**) or PCNA normalized to ENO1 (**E**), presented as relative to ENO1 (**D**) and PCNA (**E**) in WT cells. Mean ± SD, *n* = 3.

The glycolytic enzyme ENO1 is reported to be overexpressed in cancer and inflammatory cells and this promotes glycolysis, activates signalling pathways important in cancer including PI3K/AKT/mTOR, and drives tumor migration, invasion, and metastasis [33, 34]. ENO1 has the APIM consensus sequence KFAGR (422–426) in the C-termini [2]. To investigate if this is a functional PCNA interacting motif, it was fused to YFP and co-expressed with CFP-tagged PCNA. Confocal analysis showed that the KFAGR sequence fused to YFP co-localized with PCNA in replication foci. Co-localization was not detected for the F2A mutated version of the same sequence (KAAGR), a mutation known to strongly reduce APIM´s affinity to PCNA (Fig. 1A) [2, 3, 10]. This indicates that KFAGR in ENO1 is a functional APIM. Because haematological cells, both cancer and primary cells, have clear responses to treatment with APIM-peptides [3, 10], F in KFAGR in ENO1 was mutated in the haploid chronic myeloid leukaemia cell line HAP1. This led to ~90% reduction in the levels of ENO1 F423A in the two individual clones (M1 and M2) compared to the ENO1 levels in the parental cells (WT) (Fig. 1B). However, the overall cellular distribution of ENO1 F423A was similar as for ENO1 WT (Fig. 1C, left), and co-localization of PCNA and ENO1 was visible by immunofluorescence (IF) in the cytosol in both cell lines (Fig. 1C, yellow in Merged). In order to examine direct interaction between ENO1 and PCNA, the in situ Proximity ligation assay (PLA) [35] detecting if proteins are closer than 40 nm was used. The results showed positive PLA signals in the cytosol in almost all WT cells (> 99%), while the ENO1 F423A cells had the same levels of spots as the negative controls (< 10%)(Fig. 1C, right and lower panel, yellow spots). This clearly shows that the ENO1 F423A mutation abolished the ENO1 – PCNA interaction. In agreement with these results, immunoprecipitation experiments (IPs) revealed strongly reduced interactions between ENO1 F423A - PCNA compared to WT ENO1 - PCNA (Fig. 1D, E). The levels of ENO1 in cell extracts after IP were not significantly reduced compared to the input (Fig. 1B, input, compared to post IP shown in lower panels in Fig. 1D, E); thus, reduced ENO1 was not due to depletion of ENO1 in these extracts. Altogether these observations show that PCNA binds ENO1 via APIM, and that this interaction increases the cellular ENO1 protein levels.

**Fig. 2:**
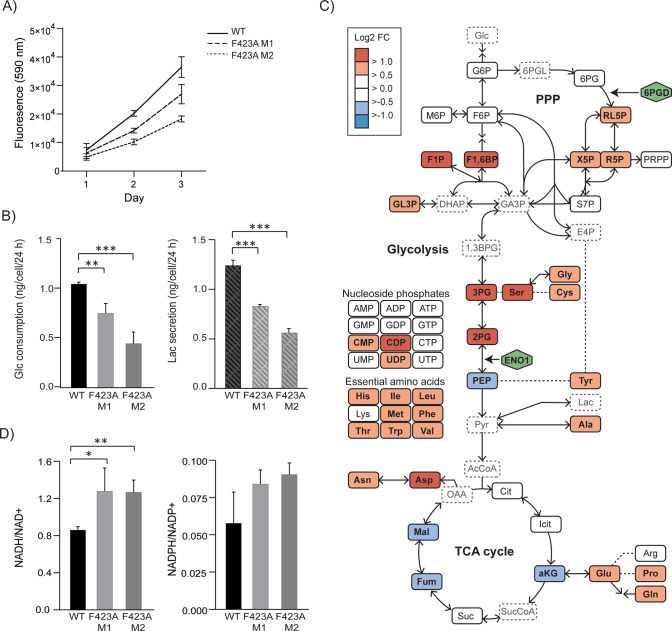
Mutation of APIM in ENO1 reduces cell growth and alters carbon flux and central carbon metabolite pools. **A** Growth of parental (WT) and ENO1 F423A mutant (M1 and M2) HAP1 cells measured over 3 days. Mean ± SD, *n* = 3. **B** Glucose consumption and lactate secretion (ng per cell per 24 h) in WT and F423A M1 and M2 cells. Mean ± SD, *n* = 4. **C** Simplified schematic overview of central carbon metabolism. Arrows indicate reactions and possible directionalities. Dashed lines link precursor and product metabolite in multistep reactions. Log2 fold changes (FC) of metabolite levels in F423A M2 cells relative to WT cells are heat mapped. Mean from 3 repeated experiments with 3 replicate cultures each are shown (*n* = 9). Dashed outlines indicates that the metabolite is not covered by the analytical method. The non-essential amino acids are linked to their precursor metabolite while the essential amino acids are presented in the left panel. **D** Ratios of NADH/NAD^+^ and NADPH/NADP^+^ in WT and F423A M1 and M2 cells. Mean ± SD, *n* = 3. Experimental details are listed in Supplementary Table S1, and metabolite abbreviations with HMDB IDs are listed in Supplementary Table S2A. A complete list of log2 FC relative to WT are listed for all metabolites and repeated experiments in Supplementary Table S2B.

Next, the phenotypic consequences of impairing the PCNA - ENO1 interaction were investigated. Both clones of ENO1 F423A cells (M1 and M2) grew slower than the parental (WT) cells (Fig. 2A). They also consumed less glucose and secreted less lactate than WT cells (Fig. 2B). To explore if this was due to the low protein levels of ENO1 in the mutant cells, which may cause a bottleneck in the glycolysis, quantitative mass spectrometric metabolite profiling of central carbon metabolism was performed. PPP intermediates and glycolytic intermediates upstream of ENO1 accumulated in ENO1 F423A cells, while levels of the downstream metabolites, including PEP and tricarboxylic acid (TCA) cycle intermediates, were reduced compared to WT (Fig. 2C, Supplementary Table S2A, B). Both essential and non-essential amino acids accumulated in the mutant cells (Fig. 2C). Nucleoside phosphate pools were mostly unaffected, and consequently, so was the adenylate energy charge (ratio ATP vs. ADP and AMP), which reflects the cells’ ability to run energy-consuming reactions [23]. The NADH/NAD^+^ and NADPH/NADP^+^ redox ratios were increased in the mutant cells (Fig. 2D). NAD^+^ is needed to run glycolysis, but the shift in NADH/NAD + did not appear to have negative metabolic consequences as the energy charge were maintained. The increased NADPH/NADP^+^ ratio indicates sufficient reducing power for the biosynthesis of fatty acids and nucleic acids. Therefore, no evident limitations were found at the carbon precursor nor energetic supply levels that explains the reduced growth rates in mutants versus WT cells. However, significant adaptions at carbon metabolite and flux levels were measured because of impairment of the ENO1 – PCNA interaction.

### Destabilizing ENO1 activates oxidative phosphorylation and reduces activation of AKT

**Fig. 3:**
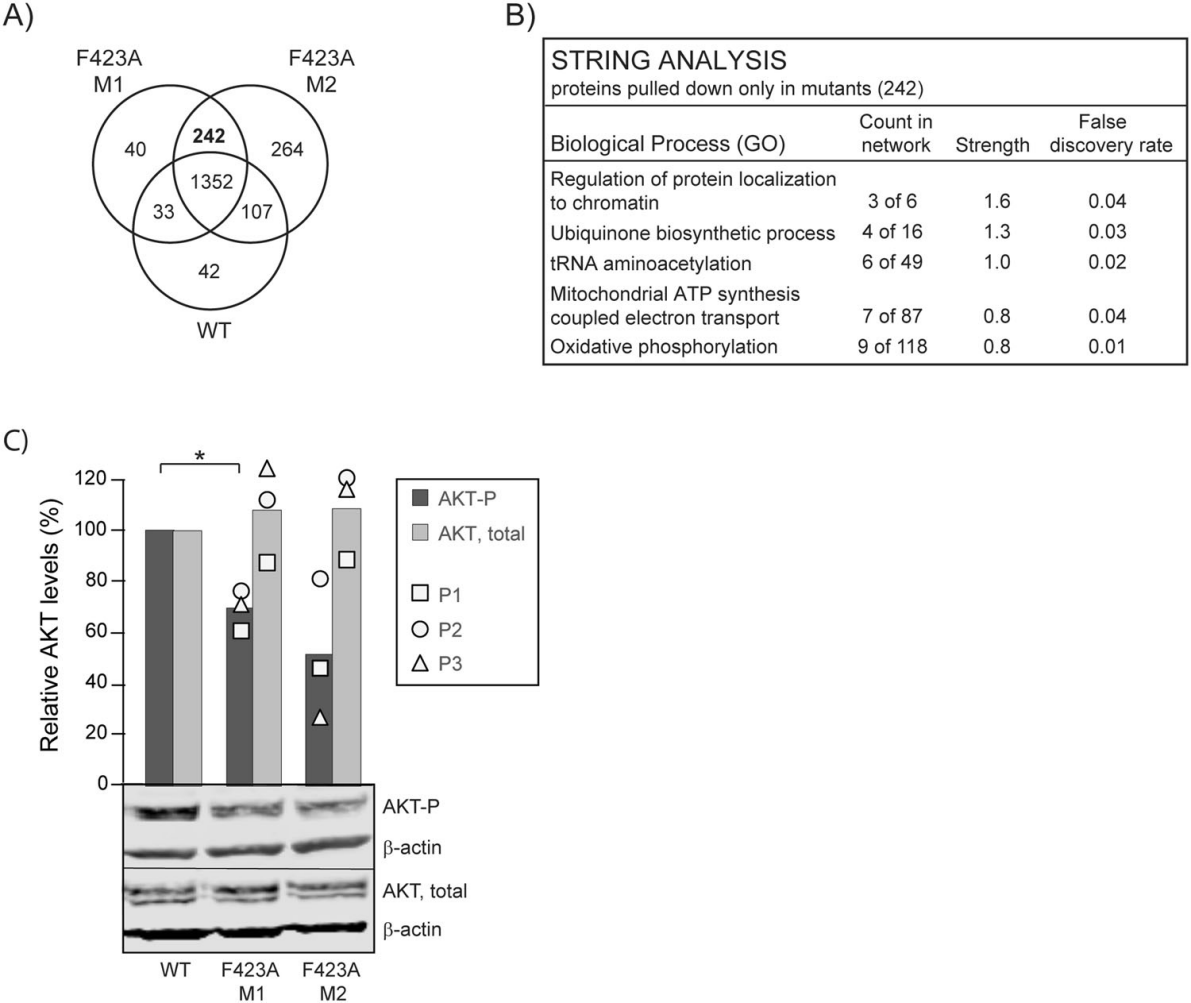
Mutation of APIM in ENO1 activates oxidative phosphorylation and reduce activation of AKT. **A** Venn diagram of proteins pulled down from cell extracts of parental (WT) and ENO1 F423A (M1 and M2) HAP1 cells using the multiplexed inhibitor bead (MIB)-assay. Only proteins detected in pull downs from all experiments (*n* = 3) are included. **B** Summary of STRING network analysis of the 242 proteins pulled down only from F423A M1 and M2 extracts. **C** Lower panel: representative western blot showing p-Ser 473 AKT, β-actin and total AKT in WT and F423A M1 and M2 extracts. Upper panel: densitometric quantifications of p-Ser 473 AKT and total AKT levels, both normalized to β-actin, presented relative to ENO1 levels in parental WT cells. Mean ± SD, *n* = 3, quantification of the individual replicas is shown as square, circle and triangle. *p < 0.05, paired two-tailed student *t*-test.

Rewiring of cellular metabolism due to hyperactivation of signalling pathways is a well described hallmark of cancer [36]. The activation of cellular anabolism is necessary to provide cells with sufficient building blocks for rapid growth. However, activation of anabolism has also been shown to promote cellular proliferation and tumorigenesis in absence of additional oncogenic transforming events [37]. Interestingly, elevated ENO1 levels are shown to activate growth and migration in cancer cells via activation of multiple signalling pathways [38, 39]. This illustrates the two-way interconnectedness of metabolism and cellular signalling. Therefore, the effect of the ENO1 F423 A mutation on cellular signalling was next explored by standard western blot analysis and the mass spectrometry (MS)-based multiple inhibitory bead (MIB)-assay. The MIB-assay is based on pulling down activated signalling proteins and/or complexes via their increased affinity to kinase inhibitors [27, 40]. Using this approach, 242 unique proteins were pulled down only from the mutant cell lines, i.e., these proteins were activated (Fig. 3A). 54 of these proteins were connected to mitochondrial functions including 15 NADH dehydrogenases, out of which five were members of the respiratory complex I (A2, A4, A6, V3, and S2)(Supplementary Table S3). STRING analysis of these 242 proteins supported increased activation of proteins involved in mitochondrial ATP synthesis coupled electron transport and oxidative phosphorylation (Fig. 3B). This possibly links to the increased NADH/NAD^+^ ratio observed in the mutant cell lines (Fig. 2D), as high NADH levels might activate oxidative phosphorylation for re-oxidation to NAD^+^. However, neither STRING analysis of proteins in the different subgroups of the Venn diagram, nor exploring whether proteins were activated or deactivated in mutant versus WT cells (including those found in one group but not in the others) could explain why the mutants grew slower than WT cells. Because ENO1 previously is reported to stimulate AKT via several pathways [38, 39, 41], AKT phosphorylation was next examined directly by western blot analysis. This revealed that the mutant cells had 30–50% lower levels of p-Ser 473 AKT compared to WT cells, while total AKT levels were the same (Fig. 3C). This is in line with previous reports showing reduced AKT activation after knockdown of ENO1 [42]. In sum, the F423A mutation in ENO1 impaired the PCNA interaction, reduced the ENO1 levels, activated mitochondrial metabolism and reduced activation of AKT. The latter likely explains the reduced growth rates detected for the mutant cells.

### Targeting PCNA with the APIM-containing peptide ATX-101 reduces glycolytic flux and alters metabolite pool composition

**Fig. 4:**
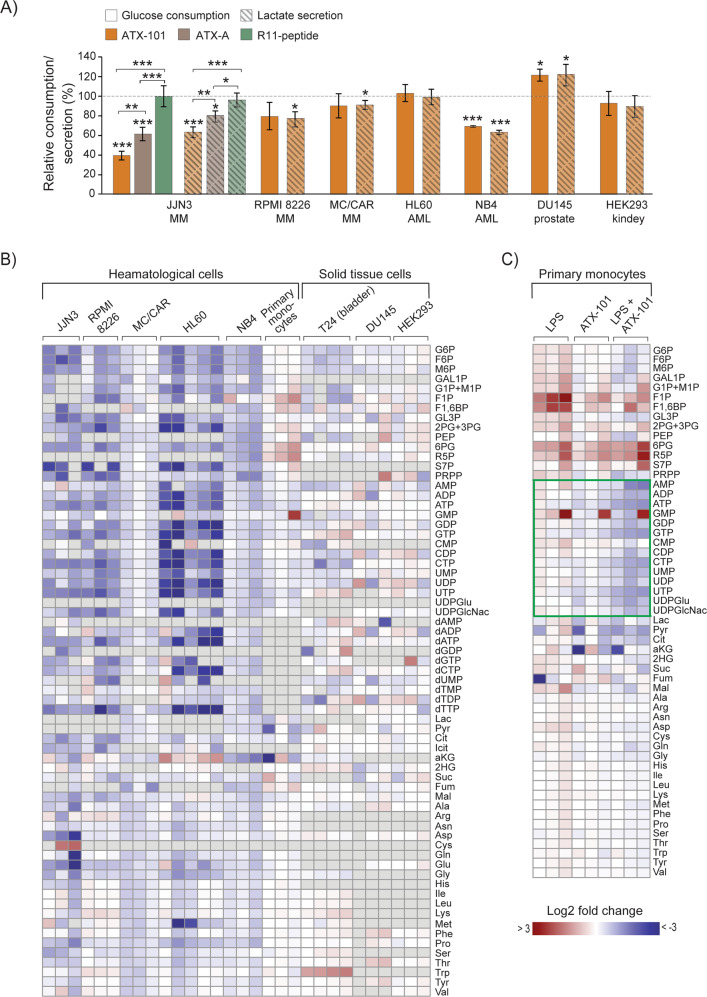
Targeting protein - PCNA interactions in haematological cells reduced central carbon metabolite pools. **A** Glucose consumption (solid bars) and lactate secretion (dashed bars) per cell per 24 h given relative to untreated control (mean ± SD) in JJN3 cells (MM) treated with ATX-101 (orange), ATX-A (brown), or R11-peptide (green) (all 8 µM) (left) and ATX-101 treated MC/CAR (MM, 8 µM), RPMI 8226 (MM, 8 µM), NB4 (AML, 8 µM), HL60 (AML, 8 µM), HEK293 (embryonic kidney, 10 µM), and DU145 (prostate cancer, 8 µM) cells. (JJN3: *p < 0.05, **p < 0.01, ***p < 0.001, ANOVA, post hoc Tukey’s test, *n* ≥ 4, Other cell lines: *p < 0.05, **p < 0.01, ***p < 0.001, unpaired two-tailed student *t*-test, *n* ≥ 3). **B** Heat mapped log2 fold change of all quantified central carbon metabolites in ATX-101 treated cells given relative to untreated control; JJN3, RPMI 8226, MC/CAR, HL60, NB4, primary monocytes (all 8 µM ATX-101, 4 h), T24 (bladder cancer, 16 µM ATX-101, 24 h), DU145 (8 µM ATX-101, 4, 8, 24 h) and HEK293 (8 µM ATX-101, 4, 8, 24 h). **C** Heat mapped log2 fold change of central carbon metabolites in primary monocytes from three donors for LPS (10 ng/ml), ATX-101 (8 µM) (same data as monocytes in (**B**) and the combination treatment relative to untreated control. **B**, **C** Grey colour in heat map = not measured. Mean ± SD, *n* ≥ 3. Absolute Log2 fold changes are listed in Supplementary Table S2C. Experimental details are listed in Supplementary Table S1 and in [9]. Metabolite abbreviations and HMDB IDs are listed in Supplementary Table S2A. These results are previously published in BioRxiv [49].

Because mutation of APIM in ENO1 significantly reduced glucose consumption and lactate secretion, potential metabolic effects of inhibiting PCNA - protein interactions using the experimental peptide drug ATX-101 containing APIM [3] was next examined. This approach allows exploration of the parallel inhibition of multiple PCNA – protein interactions at the cellular level, as opposed to changing one PCNA interacting motif in one protein. Initially, different cell lines were screened for the effect of ATX-101 treatment on glucose consumption and lactate secretion. Reductions in respectively two and four out of seven cancer cell lines were detected; thus, no unambiguous trend was found (Fig. 4A). The cell line responding with largest reductions, the MM cell line JJN3, was also treated with an ATX-101 variant with strongly reduced affinity for PCNA [3, 10], ATX-A, and a peptide containing only the cell-penetrating part, R11-peptide. These treatments resulted in significantly less, or no changes in glucose consumption, respectively (Fig. 4A, left). This confirms that the effects of ATX-101 were linked to the peptide´s affinity to PCNA. Next, the effects of ATX-101 treatment on primary metabolite pools were investigated in the same cell lines as in Fig. 4A, in addition to one bladder cancer cell line and primary monocytes from three donors. While the haematological cancer cell lines (MM and AML) responded to ATX-101 treatment with a reduction in most glycolytic and PPP intermediates, nucleoside phosphates and amino acids (Fig. 4B, JJN3 - NB4), fewer, and less distinct changes were found in primary monocytes and cell lines originating from solid tissue (Fig. 4B, right part). However, ATX-101 treatment in combinations with activation of the monocytes by lipopolysaccharide (LPS) induced a prominent reduction in the nucleoside phosphate pools (Fig. 4C, green frame). This suggests that the ATX-101 induced metabolic shift may depend on cellular stress levels, which is in accordance with the increased affinity of APIM for PTM-modified PCNA [2, 6].

The accumulation of intermediates above and depletion of intermediates below the ENO1 catalysed step in glycolysis as observed in the ENO1 F423A cell lines was not observed after ATX-101 treatment, which instead lead to a reduction in multiple metabolite pools throughout the glycolysis and PPP. This is probably a consequence of ATX-101´s ability to inhibition of multiple PCNA - proteins interactions, and thereby affecting multiple enzymes involved in both metabolism and cellular signalling. However, extensive signalome analysis did not reveal common signatures among the haematological cell lines (Supplementary Fig. S1). Still, the analysis showed that hundreds of proteins belonging to multiple signaling pathways were affected by ATX-101.

### Targeting PCNA with ATX-101 reduces both 6PGD activity and 6PGD, GAPDH and ENO1 protein levels

**Fig. 5:**
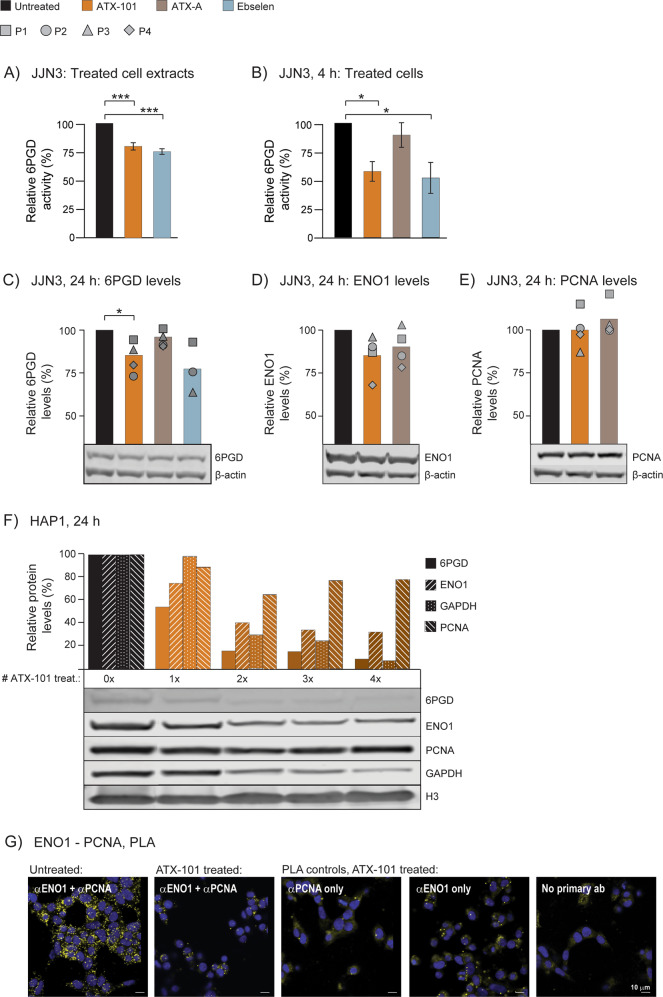
ATX-101 treatment reduces 6PGD activity and 6PGD and ENO1 protein levels and blocks ENO1 – PCNA interactions. **A–E** 6PGD activity and protein levels measured in JJN3 cells and cell extracts exposed to no (black), ATX-101 (8 μM, orange), ATX-A (8 μM, brown) and Ebselen (20 μM, blue) treatment. **A**, **B** 6PGD activity (OD = 460 nm) are plotted as relative to untreated control, mean ± SEM. (**A**) 6PGD activity in cell extracts from untreated JJN3 cells after addition of ATX-101 and Ebselen immediately before measurements, *n* = 11. (**B**) 6PGD activity in cell extracts from JJN3 cells treated with ATX-101, ATX-A and Ebselen for 4 h, *n* = 5. (**C**, **E**) Lower panels: representative western blots (WB) showing β-actin and (**C**) 6PGD, (**D**) ENO1 and (**E**) PCNA levels in JJN3 cells 24 h after ATX-101 treatment. Upper panel: densitometric quantifications of (**C**) 6PGD, (**D**) ENO1 and (**E**) PCNA levels normalized to β-actin, presented as relative to untreated controls. The mean levels and the levels in each experiments, *n* = 4 (square, circle, triangle and diamond) are shown. *p < 0.05, ***p < 0.001, two tailed, paired t-test. Experimental details are listed in Supplementary Table S1. **F** Lower panels: WB showing 6PGD, GAPDH, ENO1, PCNA and H3 (Histone 3) levels in WT HAP1 cells treated with ATX-101 (20 μM) 1-4 times (*t* = 0, 4, 8, and 12 h) and harvested at *t* = 24 h. Upper panel: densitometric quantifications of ENO1, 6PGD, GAPDH and PCNA normalized to H3 levels, presented as relative to untreated control. **G** Proximity Ligation Assay (PLA) analysis of WT HAP1 cells untreated (left image) or treated with ATX-101 (20 μM) (four right images) 5 times. Cells were probed with α-ENO1 and α-PCNA (two left images), and in the PLA controls with only α-PCNA, only α-ENO1 or no primary antibodies (ab). Positive PLA signals (yellow spots), DAPI (blue). Scale bar: 10 µm.

The metabolic effect detected after ATX-101 treatment is likely beyond the impairment of one single protein – PCNA interaction, and in addition to ENO1, the PPP enzyme 6PGD, catalysing the third step in the oxidative part of PPP (indicated in Fig. 2C), is a candidate in this context as it contains a putative PIP-box. Reduced 6PGD activity would have a direct effect on *de novo* nucleoside phosphate biosynthesis, and this could contribute to the reductions in nucleoside phosphate pools detected (Fig. 4B and highlighted in green frame in Fig. 4C). ATX-101 was found to significantly reduce 6PGD activity both when added directly to cell extracts (Fig. 5A), and in extracts made from ATX-101 treated cells (Fig. 5B). ATX-A treatment did not reduce the 6PGD activity (Fig. 5B), suggesting that the observed effect was due to inhibition of a 6PGD - PCNA interaction. Ebselen, a known inhibitor of 6PGD, reduced the activity to the same levels as ATX-101 (Fig. 5A, B). Further, a small reduction (10-20%) in 6PGD protein level was found in cell extract from ATX-101 treated cells, but not in cells treated with ATX-A (Fig. 5C). This supports that binding of 6PGD to PCNA is important for its stability; however, the reduction in 6PGD activity is rapid and not dependent on protein degradation because it was detected in cell extracts added ATX-101 (Fig. 5A) while 6PGD protein levels were significantly reduced at 24 h but not at 4 h (Fig. 5C and Supplementary Fig. S2A).

A trend towards a reduction of the ENO1 protein levels after ATX-101 treatment was also detected at 24 h but not at 4 h (Fig. 5D and Supplementary Fig. S2B, respectively). Previously, a reduction in PCNA levels after ATX-101 treatment were reported in glioblastoma cell lines [43]; however, this was not the case in JJN3 cells at the ATX-101 doses used in these experiments (Fig. 5E and Supplementary Fig. S2C). The degree of ENO1 reduction is likely dose and cell dependent, and when increasing the dose of ATX-101 in the more robust HAP-1 cells, treatment with ATX-101 caused a 25 % reduction of ENO1 levels (Fig. 5F, 1x). Like in JJN3 cells, the 6PGD levels in HAP1 cells were reduced more by ATX-101 than the ENO1 levels, and a ~40% reduction was detected already after 1x treatment. The PCNA level was less affected (~10% reduction). Because ATX-101 is both broken down by serum proteases in the media and rapidly taken up by cells, no or very low levels of ATX-101 is available after 1–2 h ([3] and unpublished). To examine how repeated treatments affected the levels of metabolic proteins, cells were treated with ATX-101 up to 4 times with 4 h intervals. This led to a dose-dependent reduction of 6PGD and ENO1 levels compared to untreated control. The levels of PCNA were also more reduced after repeated treatments, but with a maximal reduction of 30%, PCNA does not follow the same pattern as the metabolic enzymes. PCNA is previously shown to be in complex with and to stimulate the enzymatic activity of the glycolytic enzyme GAPDH [17, 19]. GAPDH does not contain any putative PCNA interacting motifs so the association with PCNA might be indirect; however, repeatable ATX-101 treatments caused a reduction also of the protein levels of GAPDH (Fig. 5F). The reduction of all these three enzymes indicate that targeting PCNA affects the stability of complexes important for metabolism, i.e., metabolons.

Next, inhibition of the ENO1 – PCNA interactions by ATX-101 was explored. The IP experiments (Fig. 1D,E) indicated that the ENO1 – PCNA interaction were reduced 80–90% by the F423A mutation, while the PLA results (Fig. 1C) suggested that the interaction was abolished. Because the PLA analysis is not quantitative and have a limited window of detection, most of the interactions therefore likely need to be blocked to detect a reduction. Cells were therefore treated 5 times with ATX-101 with 1 h intervals. The treatment reduced the cell viability ~50%, and the ENO1 level was reduced but still detectable (Supplementary Fig. S3). The level of ENO1 - PCNA interaction on the other hand, was in the ATX-101 treated cells reduced to the same level as in the PLA controls (Fig. 5G). This further supports that ATX-101 blocks the ENO1 – PCNA interaction.

## Discussion

Metabolic reprogramming is established as a hallmark of cancer [36] and several attempts have been made to attack this malignant transformation; however, no efficient inhibitors are yet clinically approved. This study shows that PCNA interactions are important for stability and activity of the metabolic enzymes ENO1, 6PGD and GAPDH, and thereby important for the regulation of glycolysis, PPP and AKT signalling. Targeting this regulatory role of PCNA may be exploited in cancer therapy.

In addition to its activities in glycolysis, ENO1 is a plasminogen receptor and a DNA binding protein [39]. The plasminogen binding motif in ENO1 is localized to residues 250–256, the DNA binding region is localized to residues 97–237, and the catalytic activity to residues 36–43, 156–162, and 262–270 [33, 39, 44]. Because F423A is more than 150 residues downstream of these regions and the overall cellular localization of ENO1 F423A is like wild type ENO1, it is unlikely that the F423A mutation affects the overall protein structure and thereby the other activities of ENO1. Therefore, the most likely explanation for the biological effects of the F423A mutation in ENO1 is reduced protein stability when binding to PCNA is impaired.

Cytosolic roles of PCNA are debated, and a major challenge is to experimentally distinguish its cytosolic roles from its nuclear roles. More than 90 kinases contain a potential PCNA-interacting motif including multiple proteins upstream of AKT, e.g., two PI3K subunits, MAST3 and PLK3 contain potential APIM sequences. In addition, several proteins regulating the MEK/ERK pathway including SOS, NF1, MST4, TAOK2 and MK2 contain APIM sequences [2, 5]. Isolated PCNA complexes are reported to contain both metabolic enzymes, e.g., ENO1 [19], and proteins involved in PI3K/AKT and MAPK signalling, e.g., FAK [10]. FAK is previously shown to be activated by ENO1; however, the role of PCNA as a scaffold in this process is elusive [38]. Here we show for the first time a direct protein - protein interaction between a metabolic enzyme and PCNA. Further, we show that blocking this interaction decreases the stability of the metabolic protein, and that this alters primary metabolism and signalling.

Data presented herein and, in another study, [9], indicate that prostate, kidney, and bladder cell lines are less dependent on a PCNA-governed regulation controlling glycolysis than haematological cells. Tissue- or cell type-specific roles of PCNA and a link to glucose metabolism has been suggested by others [17, 19]; however, as we also see a metabolic shift in LPS activated monocytes, this might also be a consequence of a lower endogenous stress level in these cell lines.

ATX-101, via its APIM, interacts with the same region on PCNA as PIP-containing peptides [3, 4]. The APIM – PCNA interaction is much weaker than the canonical PIP-box – PCNA interaction in vitro, but the affinity for APIM is increased under cellular stress via PTMs on PCNA [2, 6, 10, 45]. Previously, increased inhibition of AKT by ATX-101 was reported in LPS activated monocytes [10]. Here we show that ATX-101 treatment of LPS activated monocytes lead to a more prominent reduction in the nucleoside phosphate pool than both single agent treatments. These results support that cellular stress introduced by LPS, and not only by DNA damage, as shown previously [6], increased the affinity of APIM to PCNA. ATX-101 reduces production of pro-inflammatory cytokines [10], reduce glycolysis, AKT signalling and 6PGD-activity in cancer cells or stressed cells of haematological origin (this study). Inhibition of 6PGD-activity is by others shown to reprogram CD8 + T cell metabolism, increase the T-cell effector functions and lead to higher tumoricidal activity [46]. Because immune cells close to and/or infiltrating tumors often are activated, modulation of these cells in the tumour microenvironment may be advantageous in cancer treatments. Such an effect of ATX-101 may, at least partly, explain the prolonged stable disease found in > 70% of the efficacy population in an Phase I study on advanced solid cancer patients described in detail in the back-to-back publication[13].

The aim is to explore the metabolic changes after ATX-101 treatment both in cancer tissue and in the tumor microenvironment in endogenous animal tumor models and/or in patient biopsies; however, robust quantitative measurement of metabolites from tissues is extremely challenging due to rapid turnover thus, extensive protocol developments are needed.

We are only beginning to gain knowledge about the non-canonical roles of PCNA, but recent results, including this study, are demonstrating that the cytosolic roles of PCNA are complex and vary between different cell types [3, 8–10, 14–17,19, 47]. Here we show that APIM in ENO1 mediates interaction with PCNA in cytosol, and mutation of one amino acid in APIM strongly reduces the PCNA interaction and the ENO1 protein level. Further, our data shows that the levels and/or the activity of the metabolic enzymes ENO1, GAPDH and 6PGD are reduced if PCNA are targeted with the APIM-containing peptide ATX-101. ATX-101 likely blocks multiple proteins – PCNA interactions in vivo, and here we show that ATX-101 block the ENO1 – PCNA interactions. A role of PCNA as a scaffold important for regulation of metabolism is further manifested with the large alterations at metabolite and flux levels in ATX-101 treated cells. Altogether the results presented herein supports that PCNA can serve as a scaffold in glycolytic metabolons and shows that targeting PCNA and its regulatory roles during cellular stress may be highly relevant for cancer treatment.

## Supplementary information

Supplementary Figure S1

Supplementary Figure S2

Supplementary Figure S3

Supplementary Table S1

Supplementary Table S2

Supplementary Table S3

## Data Availability

The MS proteomics data has been deposited to the ProteomeXchange Consortium via the PRIDE [48] partner repository with the dataset identifier, project ID PXD028314; raw data HAP1, PXD011044; raw data JJN3, and PXD017474; raw data NB4, MC/CAR, and primary monocytes. All results and program codes are in their respective repositories.
