# Supplementary material for: PCNA regulates primary metabolism by scaffolding metabolic enzymes
Source: Oncogene. 2022 Dec 23;42(8):613–24. doi: 10.1038/s41388-022-02579-1 (PMC9937922; doi:10.1038/s41388-022-02579-1)
Supplement: Supplementary file 1 — Supplementary Figure S1 [file 41388_2022_2579_MOESM1_ESM.pdf]

Supplementary Figure S1:

A)

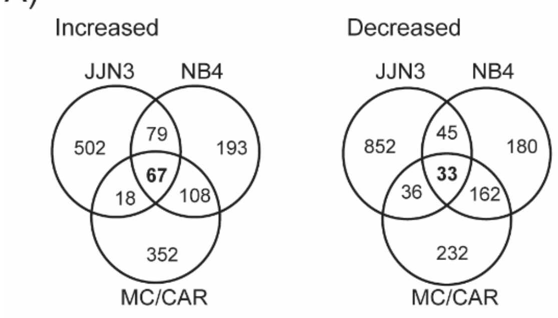

B)

Top canonical pathways, IPA: Decreased

	Proteins in pathway	P-value
1. Protein Ubiquitination Pathway	6/273	1.6E-04
2. Antigen Presenting Pathway	3/39	2.2E-04
3. Calcium Signaling	5/206	3.9E-04
4. Calcium Transport	2/10	3.9E-04
5. Unfolded Protein Response	3/56	6.4E-04

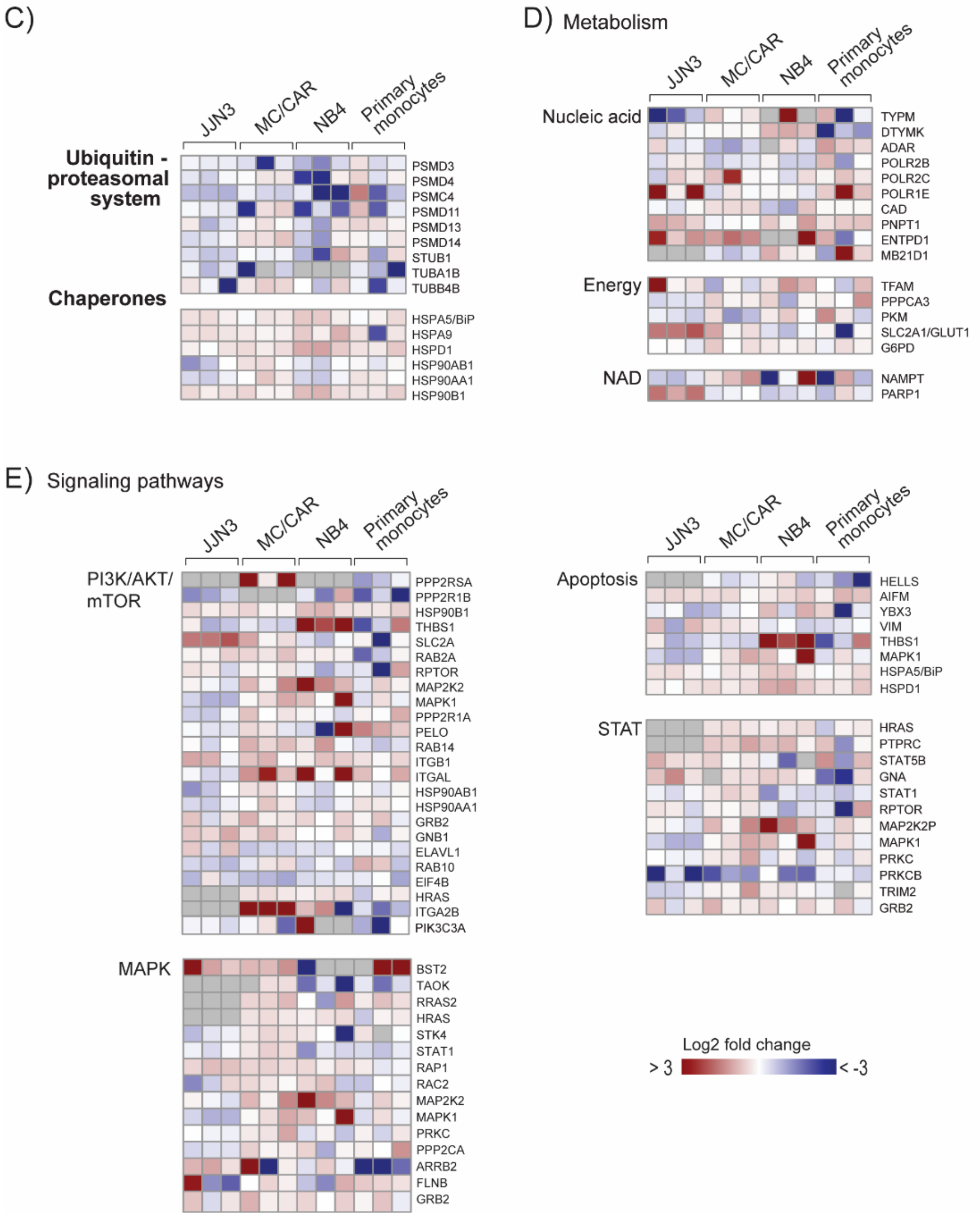

Targeting PCNA affects the regulation of protein stability, metabolism and cellular signaling in haematological cells. Changes in protein levels in haematological cells treated with ATX-101 (JJN3: 6 µM, MC/CAR, NB4, primary monocytes: 8 µM) for 4 h, as detected by the MIB-assay. (**A**) Venn diagrams displaying number of proteins significantly changed in pull downs from treated cells relative to untreated control in JJN3, MC/CAR and NB4 according to the Wilcoxon Sign Rank test. (**B**) Top canonical pathways from IPA analysis of the 33 proteins with decreased pull down levels, list and quantification of all proteins pulled down are deposit in PRIDE repository (PXD011044 and PXD017474). (**C**) Heatmaps displaying protein levels in the ubiquitin-proteasomal system and chaperones. (**D**) Heatmaps displaying protein levels involved in nucleic acid, energy, and NAD metabolism. (**E**) and the PI3K/AKT/mTOR, MAPK, apoptosis and STAT signalling pathways. Data from three repeated experiments is shown, each presented as $\log_2$ fold change relative to untreated control. Only proteins with significantly changed protein levels according to the Wilcoxon Sign Rank test in at least two cell types are shown. Grey colour in heat map = NA.

Results:

(A) Changes in the signalome after ATX-101 treatment in the three haematological cancer cell lines JJN3, MC/CAR and NB4, revealed increased pull down of 67 proteins and significantly decreased pull down of 33 proteins common to all three cell lines.

(B) INGENUITY pathways analysis (IPA) did not return any significantly enriched pathways from the list of increased proteins; however, the decreased proteins were found to be involved in protein ubiquitination, antigen presentation, calcium signaling and transport, and cellular response to unfolded protein.

(C) Pull down of multiple proteins involved in proteosomal degradation were reduced, while levels of chaperone proteins were increased, supporting changes in the dynamic regulation of protein renovation processes upon treatment with ATX-101.

(D and E) Analysis of proteins directly involved in nucleic acid and energy metabolism, PI3K/AKT/mTOR, MAPK, STAT and apoptosis pathways did not reveal any clear trends, but that multiple proteins and pathways were affected upon ATX-101 treatment.
