# Supplementary material for: PCNA regulates primary metabolism by scaffolding metabolic enzymes
Source: Oncogene. 2022 Dec 23;42(8):613–24. doi: 10.1038/s41388-022-02579-1 (PMC9937922; doi:10.1038/s41388-022-02579-1)
Supplement: Supplementary file 2 — Supplementary Figure S2 [file 41388_2022_2579_MOESM2_ESM.pdf]

Supplementary Figure S2:

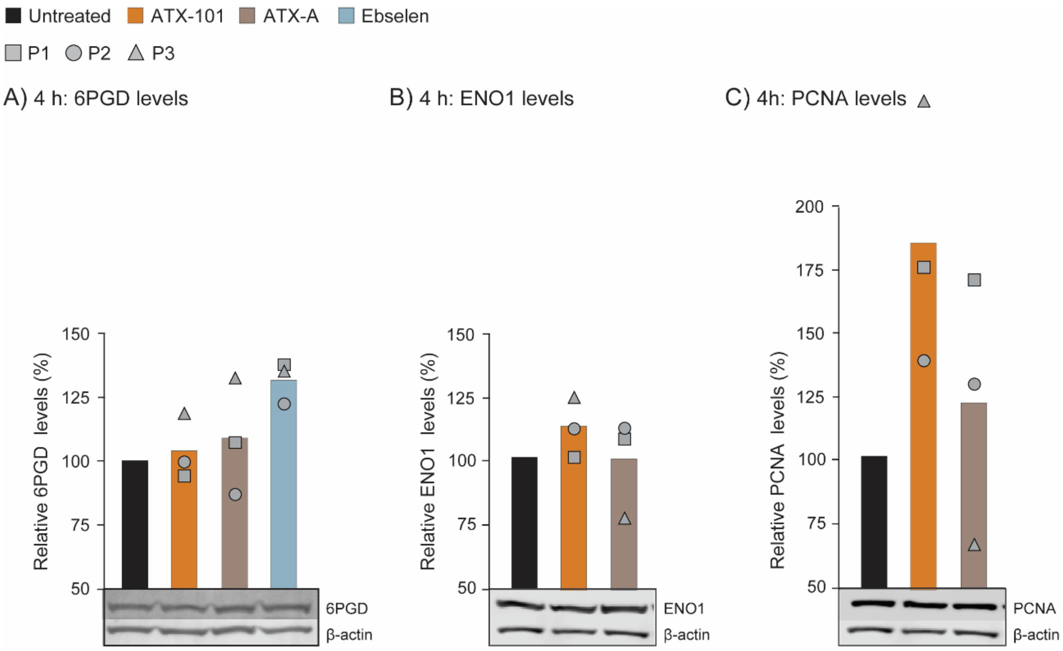

No changes in 6PGD, ENO1 or PCNA protein levels in JJN3 cells treated ATX-101 for 4 h. (A) 6PGD, **(B)** ENO1 and (**C**) PCNA levels measured by western analysis in cell extracts from JJN3 cells exposed to no treatment (black), ATX-101 (8 µM, orange). ATX-A (8 µM, brown) and the 6PGD inhibitor Ebselen (20 µM, blue) for 4 h. Densitometric quantifications of 6PGD/ENO1/PCNA levels are normalized to β-actin and presented as relative to the levels in untreated cells. The mean values are represented by bars, and the value from each of the three repeated experiments are represented with different symbols (circle, triangle and diamond). Representative western blots of 6PGD/ENO1 and β-actin are shown below the bars.

Results:

- **(A)** No reduction of 6PGD protein levels in JJN3 cells after 4 hours treatment with ATX-101.
- **(B)** No reduction of ENO1 protein levels in JJN3 cells after 4 hours treatment with ATX-101.
- **(C)** No reduction of PCNA protein levels in JJN3 cells after 4 hours treatment with ATX-101
