# Supplementary material for: PCNA regulates primary metabolism by scaffolding metabolic enzymes
Source: Oncogene. 2022 Dec 23;42(8):613–24. doi: 10.1038/s41388-022-02579-1 (PMC9937922; doi:10.1038/s41388-022-02579-1)
Supplement: Supplementary file 3 — Supplementary Figure S3 [file 41388_2022_2579_MOESM3_ESM.pdf]

Supplementary Figure S3:

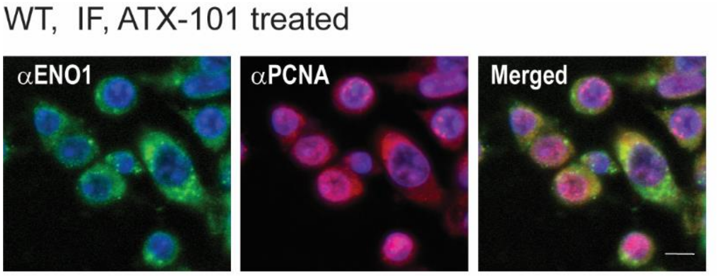

ENO1 levels in ATX-101 treated cells. Immunofluorescence (IF): confocal images of endogenous levels of ENO1 and PCNA in HAP1 parental cells (WT) treated with ATX-101 (20 μM) 5 times. Fixed cells were probed with α-ENO1 (green) and α-PCNA (red), respectively, and the nuclei are stained with DAPI. Scale bar is 10 μm.

Results:

The cells contain visible ENO1 also after repeated ATX-101 treatments. The spots detected are likely unspecific interaction with full length ATX-101 as the amounts are reduced from 1-4 hour after last treatment as the peptide is degraded.
