# Supplementary material for: PCNA regulates primary metabolism by scaffolding metabolic enzymes
Source: Oncogene. 2022 Dec 23;42(8):613–24. doi: 10.1038/s41388-022-02579-1 (PMC9937922; doi:10.1038/s41388-022-02579-1)
Supplement: Supplementary file 4 — Supplementary Table S1 [file 41388_2022_2579_MOESM4_ESM.pdf]

Supplementary Table S1: Cell densities, treatment concentrations, number of replicate cell cultures and repeated experiments, and sampling time points listed for all individual assays and cell lines/types

		Stressor concentration						Number of replicates		
**Assay, cell line**	**Cell density (cells/ml)**	**ATX-101 (µM)**	**ATX-A (µM)**	**R11-peptide (µM)**	**LPS (ng/ml)**	**ATX-101 (µM) + LPS (ng/ml)**	**Ebselen (µM)**	**Replicate cell cultures (n)**	**Repeated experiments**	**Time of harvest (h)**
**WB**										
HAP1 WT, M1, M2	500 000							1	3,4	
JJN3	500 000	8	8				20	1	3,4	4,24
**6PGD activity**										
JJN3 (treated cells)	500 000	8	8				20	1	5	4
JJN3 (treated extract)		8					20	1	11	Directly
**LC-MS/MS analysis of pyridine nucelotides**										
HAP1 WT, M1, M2	1500000, 900000, 900000	10						3	2	24
**Viability assay**										
HAP1 WT, M1, M2	30,000	-						4	3	24, 48, 72
**Quantification of extracellular metabolites**										24
HAP1 WT, M1, M2	1500000, 900000, 900000	10						3	4	
JJN-3	500 000	8	8	8				4-6	1	
DU145	70% confluent	8						3	2	
Hek293	70% confluent	10						3	2	
RPMI 8226	500 000	8						4-6	1	
MC/CAR	500 000	8						4	3	
HL60	500 000	8						4-6	1	
NB4	500 000	8						4	3	
**Targeted mass spectrometric metabolite profiling**										
HAP1 WT, M1, M2	1500000, 900000, 900000							3	3	24
JJN-3	500 000	8						3-5	3	4, 8, 24
DU145	50% confluent	8						1	1	4, 8, 24
Hek293	50% confluent	8						1	1	4
RPMI 8226	500 000	8						3	1	4
MC/CAR	500 000	8						4	3	4
HL60	500 000	8						5	1	4
NB4	500 000	8						4	3	4
Primary monocytes	4 000 000*	8			10	8 + 10		3	3	4
**Multiplexed inhibitor bead (MIB)-assay**								1	3	4
HAP1 WT, M1, M2	400,000 - 600,000	10								
JJN-3	150 000	6								
MC/CAR	500 000	8								
NB4	500 000	8								
Primary monocytes	4 000 000*	8								

*Total number of mononucleated cells seeded
