# Supplementary material for: PCNA regulates primary metabolism by scaffolding metabolic enzymes
Source: Oncogene. 2022 Dec 23;42(8):613–24. doi: 10.1038/s41388-022-02579-1 (PMC9937922; doi:10.1038/s41388-022-02579-1)
Supplement: Supplementary file 5 — Supplementary Table S2 [file 41388_2022_2579_MOESM5_ESM.pdf]

Supplementary Table S2A: Metabolite abbreviations and HMDB ID's

Abbreviation	Common name	HMDB ID
2PG+3PG	2-Phospho-D-glycerate	HMDB0000807
	3-Phospho-D-glycerate	HMDB0000362
2HG	2-Hydroxyglutarate	HMDB0059655
6PG	6-Phospho-D-gluconate	HMDB0001316
ADP	Adenosine diphosphate	HMDB0001341
aKG	a-Ketoglutaric acid	HMDB0000208
Ala	L-Alanine	HMDB0000161
AMP	Adenosine monophosphate	HMDB0000045
Arg	L-Arginine	HMDB0000517
Asn	L-Asparagine	HMDB0000168
Asp	L-Aspartic acid	HMDB0000191
ATP	Adenosine triphosphate	HMDB0000538
CDP	Cytidine diphosphate	HMDB0001546
Cit	Citric acid	HMDB0000094
CMP	Cytidine monophosphate	HMDB0000095
CTP	Cytidine triphosphate	HMDB0000082
Cys	L-Cysteine	HMDB0000574
dADP	Deoxyadenosine diphosphate	HMDB0001508
dAMP	Deoxyadenosine monophosphate	HMDB0000905
dATP	Deoxyadenosine triphosphate	HMDB0001532
dCTP	Deoxycytidine triphosphate	HMDB0000998
dGDP	Deoxyguanosine diphosphate	HMDB0000960
dGMP	Deoxyguanosine monophosphate	HMDB0001044
dGTP	Deoxyguanosine triphosphate	HMDB0001440
dTDP	Thiamine diphosphate	HMDB0001274
dTMP	Thiamine monophosphate	HMDB0002666
dTTP	Thymidine triphosphate	HMDB0001342
dUMP	Deoxyuridine monophosphate	HMDB0001409
dUTP	Deoxyuridine triphosphate	HMDB0001191
F1,6BP	Fructose 1,6-bisphosphate,	HMDB0001058
F1P	Fructose 1-phosphate	HMDB0001076
F6P	Fructose 6-phosphate	HMDB0000124
Fum	Fumaric acid	HMDB0000134
G1P+M1P	Glucose 1-phosphate	HMDB0001586
	Mannose 1-phosphate	HMDB0006330
G6P	Glucose 6-phosphate	HMDB0001401
GA3P	Glyceraldehyde 3-phosphate	HMDB0001112
GAL1P	Galactose 1-phosphate	HMDB0000645
GAL6P	Galactose 6-phosphate	-
GDP	Guanosine diphosphate	HMDB0001201
GL3P	Glycerol 3-phosphate	HMDB0000126
Gln	L-Glutamine	HMDB0000641
Glu	L-Glutamic acid	HMDB0000148
Gly	Glycine	HMDB0000123
GMP	Guanosine monophosphate	HMDB0001397
GTP	Guanosine triphosphate	HMDB0001273
His	L-Histidine	HMDB0000177
ICit	Isocitric acid	HMDB0000193
Ile	L-Isoleucine	HMDB0000172
Lac	L-Lactic acid	HMDB0000190
Leu	L-Leucine	HMDB0000687
Lys	L-Lysine	HMDB0000182
M6P	Mannose 6-phosphate	HMDB0001078
Mal	L-Malic acid	HMDB0000156
Met	L-Methionine	HMDB0000696
NAD^+	Nicotinamide Adenine Dinucleotide oxidized	HMDB0000902
NADH	Nicotinamide Adenine Dinucleotide reduced	HMDB0001487
$NADP^+$	Nicotinamide Adenine Dinucleotide phosphate oxidized	HMDB0000217
NADPH	Nicotinamide Adenine Dinucleotide phosphate reduced	HMDB0000221
PEP	Phosphoenolpyruvic acid	HMDB0000263
Phe	L-Phenylalanine	HMDB0000159
Pro	L-Proline	HMDB0000162
PRPP	Phosphoribosyl pyrophosphate	HMDB0000280
Pyr	Pyruvic acid	HMDB0000243
R5P	D-Ribose 5-phosphate	HMDB0001548
S7P	D-Sedoheptulose 7-phosphate	HMDB0001068
Ser	L-Serine	HMDB0000187
Suc	Succinic acid	HMDB0000254
Thr	L-Threonine	HMDB0000167
Trp	L-Tryptophan	HMDB0000929
Tyr	L-Tyrosine	HMDB0000158
UDP	Uridine diphosphate	HMDB0000295
UDP-Glc-NAc	Uridine diphosphate-N-acetylglucosamine	HMDB0000290
UDP-glu	Uridine diphosphate glucose	HMDB0000286
UMP	Uridine monophosphate	HMDB0000288
UTP	Uridine triphosphate	HMDB0000285
Val	L-Valine	HMDB0000883

Supplementary Table S2B: Log2 fold change of all measured intracellular central carbon metabolites in HAP1 M1 and M2 cells relative to HAP1 WT cells. The averages of n = 3 individual cell cultures from 2-3 repeated experiments are shown. Details on cell density and replicate numbers are listed in Supplementary Table S1. Metabolite abbreviations and HMDB IDs are listed in Supplementary Table S2A.

Pathway/ metabolite class	Metabolite	HAP1 M1			HAP1 M2		
Glycolysis, PPP, and associated phosphorylated sugars	G6P	0,1	0,0	0,6	0,4	0,1	0,6
	F6P	0,0	0,0	0,5	0,2	0,1	0,5
	M6P	0,1	-0,1	0,3	0,5	0,1	0,4
	F1,6BP	1,3	2,5	2,4	1,8	2,1	1,5
	F1P	0,8	0,9	1,1	1,0	1,0	0,9
	GL3P	-0,2	-0,4	0,1	0,6	0,2	0,6
	2PG+3PG	1,9	1,7	1,8	2,0	1,5	1,8
	PEP	-1,0	-0,4	-0,3	-0,8	-1,5	-0,1
	6PG	0,1	0,1	0,2	0,1	-0,3	-0,1
	R5P+RL5P+X5P	0,4	0,6	0,6	0,6	0,6	0,7
	PRPP	-0,2	-0,4	0,2	0,5	-0,4	0,4
	S7P	0,0	-0,5	-0,2	0,4	-0,5	-0,2
Nucleoside phosphates	AMP	-0,1	0,1	0,3	0,3	0,1	0,4
	ADP	0,2	0,5	0,3	0,4	0,2	0,5
	ATP	0,0	-0,2	0,1	0,3	0,1	0,1
	GMP	0,5	-0,1	0,6	0,5	0,0	0,7
	GDP	0,0	0,2	0,2	0,3	0,0	0,5
	GTP	-0,2	-0,3	0,1	0,1	-0,2	0,0
	CMP	-0,2	-0,1	-0,1	0,6	0,5	0,8
	CDP	0,6	0,8	0,4	1,4	0,7	0,9
	CTP	0,0	0,3	0,3	0,2	0,5	0,2
	UMP	-0,1	0,2	0,1	0,3	0,5	0,1
	UDP	0,3	0,9	0,4	1,0	0,6	0,9
	UTP	-0,1	0,1	0,2	0,2	0,4	0,2
Pyridine nucleotides	NAD^+	-	0,1	-0,3	-	-0,5	-0,8
	NADH	-	0,2	0,3	-	-0,2	-0,3
	$NADP^+$	-	0,5	-0,1	-	0,1	-0,7
	NADPH	-	0,8	0,0	-	0,7	0,0
TCA cycle	Fum	-0,8	-0,5	-0,5	-0,8	-0,9	-0,9
	Suc	0,3	0,0	1,2	0,6	-0,2	0,2
	Mal	-0,7	-0,4	-0,3	-0,7	-0,6	-0,5
	aKG	-0,8	-0,1	-0,2	-0,6	-0,5	-0,6
	Cit	-0,3	0,2	1,6	0,1	0,2	0,3
	Icit	-0,8	0,1	0,1	-0,4	0,1	0,1
Amino acids	Gly	-	-0,3	0,1	-	0,6	0,8
	Ala	-	-0,1	0,2	-	0,4	0,6
	Ser	-	0,5	0,7	-	1,0	1,0
	Pro	-	0,3	0,5	-	0,8	0,8
	Val	-	0,0	0,5	-	0,4	0,7
	Thr	-	0,1	0,4	-	0,5	0,6
	Cys	-	0,2	0,6	-	0,6	0,6
	Ile	-	0,0	0,5	-	0,4	0,6
	Leu	-	0,1	0,5	-	0,4	0,6
	Asn	-	0,2	0,4	-	0,7	0,7
	Asp	-	0,9	1,1	-	1,1	1,2
	Gln	-	0,2	0,4	-	0,5	0,6
	Glu	-	0,4	0,5	-	0,8	0,7
	Met	-	0,1	0,5	-	0,5	0,7
	His	-	0,1	0,5	-	0,5	0,6
	Phe	-	0,0	0,5	-	0,4	0,7
	Arg	-	0,0	0,4	-	0,3	0,5
	Tyr	-	0,1	0,5	-	0,4	0,6
	Trp	-	0,0	0,6	-	0,5	0,8
	Lys	-	-0,2	0,3	-	0,2	0,5

Log2 fold change

- ≥ 1.0
- ≥ 0.5
- ≤ -0.5
- ≤ -1.0

Supplementary Table S2C: Log_2 fold change of all measured intracellular central carbon metabolites in ATX-101 treated (8 µM) cells relative to untreated control cells listed for JJN3, RPMI 8226, MC/CAR, HL60, NB4, primary monocytes, DU145 and Hek293. Each of n ≥ 3 from one (RPMI8226, HL60, T24, DU145, and Hek293), or the average of n ≥ 3 from three (JJN3, MC/CAR, NB4, monocytes) repeated experiments is shown. Details on cell densities and exact replicate numbers are listed in Supplementary Table S1. Metabolite abbreviations and HMDB IDs are listed in Supplementary Table S2A.

Pathway/ metabolite class	Metabolite	JJN3			RPMI 8226			MC/CAR			HL60					NB4			Primary monocytes			DU145			Hek293		
Glycolysis, PPP and associated phosphorylated sugars	G6P	-1,5	-1,0	-1,6	-0,5	-1,3	-1,1	-0,5	-0,2	-0,1	-1,3	-1,8	-0,8	-1,0	-1,5	-0,7	-0,9	-1,2	0,0	0,1	0,0	-0,2	-0,3	-0,3	-0,2	-0,1	0,2
	F6P	-1,5	-2,1	-1,6	-0,1	-1,2	-0,9	-0,5	-0,2	0,0	-0,9	-1,8	-0,7	-1,2	-1,6	-0,7	-0,8	-1,2	-0,1	0,1	0,0	0,0	-0,1	-0,1	-0,3	0,0	0,2
	M6P	-1,5	-1,1	-1,3	-0,2	-1,2	-1,1	-0,5	-0,2	-0,2	-1,0	-1,6	-0,7	-1,1	-1,6	-0,6	-0,9	-1,2	-0,1	0,1	0,1	-0,6	-0,3	-0,3	-0,3	0,1	-0,4
	GAL1P	-0,9	NA	NA	-0,2	-1,0	-0,7	-1,0	0,1	-0,5	-0,1	-1,2	-0,4	-0,8	-1,0	-0,4	0,2	-1,1	-0,4	-0,1	0,4	NA	NA	NA	NA	NA	NA
	G1P+M1P	-1,1	NA	NA	-0,4	-1,3	-1,0	-0,4	-0,1	-0,4	-1,4	-1,9	-0,6	-1,0	-1,4	-0,6	-0,9	-1,1	-0,2	0,1	0,6	0,0	-0,3	-0,2	-0,1	0,2	-0,2
	F1P	NA	NA	NA	-0,6	-1,7	-1,6	-0,6	-0,3	0,2	-0,7	-1,6	0,1	-0,4	-1,5	1,0	0,1	-0,3	0,3	0,9	1,4	0,3	0,0	0,5	-0,3	0,3	-0,3
	F1,6BP	NA	-1,8	-0,8	NA	NA	NA	0,6	-0,6	-0,1	NA	NA	NA	NA	NA	-0,5	-0,9	-0,8	0,1	0,7	0,6	-0,4	0,2	0,1	0,5	-0,4	0,7
	GL3P	-0,7	-1,9	-1,6	-0,9	-1,6	-1,4	-0,4	0,0	-0,2	-2,0	-2,0	-0,7	-0,6	-1,1	-0,4	-0,6	-0,9	-0,2	0,1	-0,2	0,1	-0,9	0,6	-0,6	0,1	0,0
	2PG+3PG	-0,8	-0,9	-1,1	-1,4	-2,3	-1,4	-0,5	-0,3	-0,4	-2,7	-2,6	-0,6	-1,4	-2,4	-0,6	-0,9	-1,1	0,1	0,4	0,5	-0,1	0,1	0,6	-0,3	0,3	0,4
	PEP	NA	-0,6	-1,2	NA	NA	NA	-0,6	-0,4	-0,4	NA	NA	NA	NA	NA	-0,7	-1,1	-1,1	-0,3	0,0	0,6	NA	NA	1,1	-0,4	0,3	0,4
	6PG	-1,2	-1,3	-1,2	-0,5	-1,6	-1,2	-0,5	-0,2	-0,4	-0,9	-1,3	-0,8	-0,8	-1,4	-0,7	-1,0	-1,2	0,3	0,8	1,3	0,1	0,4	0,0	0,1	-0,1	0,3
	R5P	NA	NA	NA	NA	NA	NA	-0,4	0,0	-0,5	NA	NA	NA	NA	NA	-0,7	-1,0	-1,3	0,5	0,9	1,4	-0,3	-0,2	-0,3	-0,1	0,4	0,1
	S7P	-2,4	-1,8	NA	-2,4	NA	-2,3	-0,4	0,0	-0,3	-2,2	-2,9	-0,8	-1,3	-2,0	-0,5	-0,7	-1,0	-0,2	0,0	0,7	NA	NA	NA	NA	NA	NA
	PRPP	-1,2	-2,1	NA	-1,3	-1,9	-1,6	-0,3	-0,3	-0,3	-0,4	-0,6	-0,4	-0,5	-0,8	-0,4	-1,4	-1,4	-0,4	0,1	0,3	NA	NA	1,5	0,4	0,7	-1,1
(deoxy) Nucleoside phosphates	AMP	-0,2	0,7	-0,4	-0,4	-0,5	-0,8	-0,5	-0,1	-0,5	-1,9	NA	-0,8	-1,1	-1,6	-0,4	-0,5	-0,8	-0,2	-0,1	-0,4	0,6	-0,7	0,3	-0,4	-0,2	-0,7
	ADP	-0,7	-0,4	-1,1	-0,6	-1,2	-0,9	-0,6	-0,1	-0,5	-2,0	-2,6	-1,0	-1,4	-1,9	-0,3	-0,5	-0,9	-0,4	-0,1	-0,4	0,8	-1,3	0,0	0,3	-0,1	-0,3
	ATP	-0,9	-1,4	-1,6	-1,1	-1,6	-1,0	-0,6	-0,2	-0,5	-2,3	-2,7	-0,9	-1,4	-1,9	-0,3	-0,5	-0,9	-0,6	-0,3	-0,6	-0,1	0,1	-0,1	0,1	0,0	0,0
	GMP	-0,3	0,0	-0,6	-0,8	0,0	-0,6	-0,3	0,0	-0,4	NA	0,8	-0,6	-0,6	-0,7	-0,1	-0,2	-0,2	-0,2	-0,3	2,3	-0,2	-0,5	0,1	0,0	0,0	-0,1
	GDP	-0,9	-0,2	-1,2	-0,3	-1,4	-1,4	-0,6	-0,2	-0,5	-1,6	-3,8	-1,4	-2,5	-3,8	-0,2	-0,4	-0,8	-0,3	-0,1	-0,3	0,6	-0,6	0,3	-0,2	0,2	-0,5
	GTP	-0,9	-1,2	-1,7	-1,1	-1,7	-1,2	-0,6	-0,2	-0,5	-2,5	-3,3	-1,1	-1,6	-2,5	-0,3	-0,5	-0,8	-0,4	-0,2	-0,5	0,0	0,0	-0,1	-0,1	-0,1	0,2
	CMP	NA	-0,1	NA	-1,2	NA	NA	-0,3	-0,1	-0,3	-3,2	NA	0,9	NA	NA	0,0	-0,4	-0,7	0,1	0,1	0,2	NA	NA	NA	NA	NA	NA
	CDP	-1,0	-0,2	NA	-0,5	-1,4	-1,0	-0,4	-0,1	-0,4	-2,1	-2,9	-1,4	-1,5	-2,6	-0,1	-0,4	-0,7	-0,1	0,0	-0,2	0,8	-0,6	0,3	0,5	0,7	-0,6
	CTP	-1,3	-1,4	-1,5	-1,3	-2,0	-1,3	-0,6	-0,2	-0,4	-2,7	-3,4	-1,1	-1,6	-2,3	-0,3	-0,5	-0,9	-0,3	-0,1	-0,5	0,0	0,1	0,1	-0,1	0,2	0,1
	UMP	-0,6	-0,5	-1,1	-0,7	-1,5	-1,3	-0,5	-0,1	-0,3	-1,6	-2,1	-0,5	-0,8	-1,6	-0,2	-0,5	-0,7	-0,3	-0,1	-0,3	0,1	-0,3	0,4	-0,2	0,1	0,2
	UDP	-1,4	-0,3	-1,2	-0,9	-1,5	-1,1	-0,4	-0,1	-0,4	-2,4	-4,9	-1,3	-2,3	-2,9	-0,3	-0,4	-0,9	-0,4	-0,1	-0,3	1,2	-1,2	0,4	0,7	0,6	-0,8
	UTP	-1,4	-1,4	-1,6	-1,6	-2,1	-1,3	-0,5	-0,1	-0,4	-2,7	-3,2	-1,0	-1,5	-2,2	-0,4	-0,5	-0,9	-0,6	-0,2	-0,6	NA	NA	0,2	0,5	-0,1	-0,4
	UDP-glu	NA	NA	NA	NA	NA	NA	-0,5	-0,2	-0,4	NA	NA	NA	NA	NA	-0,3	-0,5	-0,7	-0,7	-0,1	-0,4	NA	NA	NA	NA	NA	NA
	UDP-Glc-NAc	-0,5	-0,8	-1,5	-1,2	-1,9	-1,3	-0,6	-0,1	-0,3	-2,0	-2,5	-0,4	-0,8	-1,7	-0,3	-0,6	-0,7	-0,3	0,0	-0,2	0,0	0,0	0,0	-0,2	0,1	-0,2
	dAMP	NA	0,1	-0,6	NA	NA	NA	NA	NA	NA	NA	NA	NA	NA	NA	NA	NA	NA	NA	NA	NA	0,2	NA	-2,1	NA	NA	NA
	dADP	-1,1	0,1	NA	0,6	-0,6	-0,7	-0,6	-0,3	-0,7	0,6	-0,7	-1,3	-2,0	-2,5	-0,2	-0,4	-0,7	NA	NA	NA	0,9	-1,1	0,1	0,4	0,4	-1,0
	dATP	-1,2	-1,2	-1,2	-0,6	-2,0	-1,3	-0,6	-0,2	-0,4	-1,1	-2,6	-1,2	-3,4	-3,5	-0,3	-0,4	-0,8	NA	NA	NA	-0,2	0,2	-0,2	0,1	-0,2	-0,2
	dGDP	NA	-1,5	-1,0	NA	NA	NA	NA	NA	NA	NA	NA	NA	NA	NA	NA	NA	NA	NA	NA	NA	NA	NA	NA	NA	NA	NA
	dGTP	-1,1	-0,9	NA	0,2	-1,3	-1,6	-0,7	-0,2	-0,6	0,7	-1,4	-1,9	NA	NA	-0,5	-0,5	-0,8	NA	NA	NA	NA	NA	NA	NA	1,5	-0,4
	dCTP	-1,0	-0,7	-0,6	-0,6	-1,1	-0,9	-0,5	-0,1	-0,4	0,1	-2,5	-1,5	-2,5	-2,7	-0,3	-0,5	-0,8	NA	NA	NA	-0,2	0,1	0,2	0,0	-0,2	0,1
	dUMP	-0,4	NA	NA	0,5	-1,4	-1,7	NA	NA	NA	1,1	-1,1	NA	-2,1	NA	NA	NA	NA	NA	NA	NA	0,0	-1,1	-0,3	-0,4	-0,4	-0,1
	dTMP	-0,2	-0,2	NA	0,6	-0,3	-0,4	-0,2	-0,1	-0,1	0,5	-0,2	-0,4	-0,5	-0,6	-0,5	-0,5	-0,5	NA	NA	NA	0,1	-0,1	-0,1	-0,4	0,0	0,0
	dTDP	-0,7	-0,1	-0,5	0,2	-0,6	-0,6	NA	NA	NA	0,3	-0,5	-0,6	-0,6	-0,8	-0,3	-0,4	-0,9	NA	NA	NA	1,0	-1,1	0,4	0,6	0,7	-0,6
	dTTP	-1,6	-1,2	-1,0	-1,1	-3,0	-1,6	-0,7	-0,1	-0,7	-0,8	-6,1	-2,0	-3,5	-5,3	-0,3	-0,5	-1,0	NA	NA	NA	0,0	0,2	0,2	-0,1	0,0	-0,9
TCA cycle and asisociated	Lac	NA	NA	NA	NA	NA	NA	-0,6	-0,3	-0,1	NA	NA	NA	NA	NA	-0,4	-0,3	-0,5	-0,8	0,2	-0,5	NA	0,1	-0,1	0,0	0,0	-0,2
	Pyr	NA	NA	NA	NA	NA	NA	-0,4	-0,4	-0,2	NA	NA	NA	NA	NA	-0,4	-0,4	-0,5	-1,9	-0,1	-1,0	NA	-0,1	-0,6	0,1	0,0	0,2
	Cit	-0,7	-1,4	-0,9	-0,4	-1,2	-1,3	-0,5	-0,3	-0,2	-0,5	-0,9	-0,2	-0,3	-0,7	-0,1	-0,2	-0,3	-0,3	0,0	-0,8	0,0	0,0	0,2	-0,3	0,1	0,0
	Icit	-1,0	-0,8	-0,8	-0,4	-1,5	-0,6	NA	NA	NA	0,2	-0,5	0,3	-0,2	-1,1	NA	NA	NA	NA	NA	NA	0,1	0,0	0,2	-0,2	-0,1	0,0
	aKG	0,5	-0,7	-1,2	0,6	0,1	0,2	-0,6	-0,4	-0,6	1,4	0,6	0,4	1,0	1,3	-0,9	-1,2	-1,6	-3,4	0,9	-1,0	NA	NA	NA	NA	NA	NA
	2HG	0,0	0,4	-0,6	0,1	-0,3	-0,2	NA	NA	NA	0,0	0,0	0,2	0,2	-0,2	NA	NA	NA	-0,1	0,0	-0,1	-0,9	-0,4	-0,1	-0,3	0,2	0,0
	Suc	-0,2	NA	-0,4	0,2	-0,5	-1,0	-0,6	-0,2	-0,3	-0,4	-0,4	-0,2	0,0	-0,6	-0,2	-0,3	-0,6	1,0	0,1	-0,5	-0,8	0,0	0,3	-0,7	0,3	0,4
	Fum	NA	NA	-0,5	NA	NA	NA	-1,1	-0,2	-1,4	NA	NA	NA	NA	NA	-0,8	-0,7	-0,7	0,4	0,2	0,6	NA	-0,3	-0,2	-0,1	0,0	0,2
	Mal	-0,7	-0,7	-1,2	-0,5	-1,1	-0,8	-0,5	-0,3	-0,4	-0,3	-0,3	0,2	0,3	-0,3	-0,3	-0,5	-0,8	-0,4	-0,3	-0,1	0,1	-0,2	0,2	-0,2	0,1	0,0
Amino acids	Ala	-0,6	-0,5	-1,6	-0,1	-0,3	-0,2	-0,7	-0,6	-0,3	0,0	-0,8	-0,4	-0,2	-0,3	-0,4	-0,3	-0,7	-0,2	0,1	-0,1	NA	0,4	0,5	0,0	0,1	0,0
	Arg	0,4	-1,1	0,2	0,4	0,5	0,4	-0,5	-0,5	-0,1	0,1	-0,5	-0,4	0,1	0,1	-0,4	-0,2	-0,6	0,2	0,1	-0,1	NA	NA	NA	NA	NA	NA
	Asn	-0,1	-0,6	-0,5	-0,2	-0,3	-0,3	-0,6	-0,5	-0,3	-0,4	-1,0	-0,6	-0,4	-0,3	-0,4	-0,3	-0,7	0,0	0,1	-0,1	NA	NA	NA	NA	NA	NA
	Asp	-1,2	-1,6	-2,7	-0,3	-0,3	-0,2	-0,6	-0,4	-0,4	-0,1	-0,8	-0,6	-0,2	-0,2	-0,2	-0,1	-0,4	-0,3	0,1	-0,1	NA	0,0	0,2	0,0	0,1	0,3
	Cys	NA	1,6	1,8	NA	NA	NA	NA	NA	NA	NA	NA	NA	NA	NA	NA	NA	NA	NA	NA	NA	NA	NA	NA	NA	NA	NA
	Gln	-0,1	-0,4	-3,7	-0,2	0,0	-0,1	-0,6	-0,6	-0,3	-0,4	-0,9	-0,7	-0,3	-0,3	-0,4	-0,2	-0,7	0,1	0,1	-0,2	NA	NA	NA	NA	NA	NA
	Glu	-1,1	-1,5	-3,1	-0,7	-0,7	-0,7	-0,7	-0,4	-0,4	0,7	-1,2	-0,6	-1,3	-0,6	-0,4	-0,4	-0,8	-0,2	0,0	-0,2	NA	-0,1	0,0	0,0	0,0	0,3
	Gly	-1,0	-0,3	-1,5	-0,4	-0,6	-0,5	-0,6	-0,5	-0,3	-0,7	-1,3	-1,0	-0,9	-0,8	-0,4	-0,4	-0,5	-0,3	0,0	-0,2	NA	NA	NA	NA	NA	NA
	His	-0,5	-0,6	-1,0	0,2	0,0	-0,1	-0,6	-0,6	-0,3	0,1	-0,6	-0,4	0,0	-0,1	-0,4	-0,2	-0,4	0,0	0,1	-0,1	NA	NA	NA	NA	NA	NA
	Ile	0,0	0,3	-0,8	0,2	0,1	0,1	-0,6	-0,6	-0,2	0,0	-0,6	-0,5	0,1	-0,1	-0,4	-0,2	-0,6	0,0	0,1	-0,1	NA	NA	NA	NA	NA	NA
	Leu	0,0	0,3	-0,9	0,2	0,1	0,0	-0,6	-0,6	-0,2	0,1	-0,6	-0,5	0,0	-0,1	-0,4	-0,2	-0,6	0,0	0,1	-0,1	NA	NA	NA	NA	NA	NA
	Lys	0,1	-0,4	-0,7	0,4	0,5	0,3	-0,6	-0,6	-0,2	0,3	-0,4	-0,4	0,2	0,1	-0,3	0,0	-0,4	0,1	0,1	-0,1	NA	NA	NA	NA	NA	NA
	Met	0,7	-0,8	NA	-0,2	0,2	0,1	-0,5	-0,7	-0,2	-0,3	-3,4	-2,2	-0,7	-0,7	-0,4	-0,2	-0,7	0,0	0,0	-0,2	NA	NA	NA	NA	NA	NA
	Phe	-0,1	-0,1	-0,8	0,2	0,1	0,0	-0,6	-0,6	-0,2	0,0	-0,7	-0,5	0,0	-0,1	-0,3	-0,2	-0,5	0,0	0,1	-0,1	NA	0,5	0,7	0,0	0,1	0,2
	Pro	-0,7	-0,8	-1,5	-0,4	-0,3	-0,3	-0,6	-0,5	-0,3	-0,7	-1,3	-0,9	-0,7	-0,7	-0,5	-0,5	-0,9	0,0	0,1	-0,1	NA	0,3	0,2	-0,2	0,0	0,0
	Ser	-1,1	-0,9	-0,5	0,0	-0,4	-0,3	-0,6	-0,4	-0,3	0,0	-0,7	-0,6	-0,2	-0,3	-0,2	-0,2	0,0	-0,2	0,1	-0,1	NA	NA	NA	NA	NA	NA
	Thr	-0,5	-0,6	-0,6	0,0	-0,2	-0,2	-0,6	-0,5	-0,3	-0,2	-0,9	-0,5	-0,3	-0,3	-0,4	-0,2	-0,5	0,0	0,1	-0,2	NA	0,6	0,8	0,1	0,1	0,2
	Trp	-0,1	-0,1	-0,5	0,4	0,3	0,2	-0,6	-0,7	-0,2	0,1	-0,6	-0,4	0,0	0,1	-0,2	0,0	-0,4	0,2	0,0	-0,3	NA	NA	NA	NA	NA	NA
	Tyr	-0,2	-0,3	-0,7	0,1	0,1	0,1	-0,6	-0,6	-0,2	0,0	-0,6	-0,4	0,0	-0,1	-0,4	-0,2	-0,5	0,1	0,1	-0,2	NA	0,3	0,6	-0,1	0,0	0,2
	Val	-0,1	0,6	-0,9	0,2	0,1	0,1	-0,6	-0,6	-0,2	0,1	-0,6	-0,4	0,1	0,0	-0,3	-0,2	-0,5	0,1	0,1	-0,1	NA	NA	NA	0,1	0,1	0,2

Log2 fold change	
(orange)	≥ 1.0
(light orange)	≥ 0.5
(white)	
(light blue)	≤ -0.5
(blue)	≤ -1.0
