# Supplementary material for: PCNA regulates primary metabolism by scaffolding metabolic enzymes
Source: Oncogene. 2022 Dec 23;42(8):613–24. doi: 10.1038/s41388-022-02579-1 (PMC9937922; doi:10.1038/s41388-022-02579-1)
Supplement: Supplementary file 6 — Supplementary Table S3 [file 41388_2022_2579_MOESM6_ESM.pdf]

Supplementary Table S3:

Mitochondrial proteins pulled down with the MIB-assay from HAP1 ENO1 F423A cells (M1 and M2), but not from parental cells (WT

Their connetion to mitochondrial functions are found using the UniprotbR package and map to the GO terms and then a simple couting using grep available within R. Only proteins pulled down in each cell line in all three repeated experiments are included.

ID	M1	M2	WT	Fasta	Gene. ontology.IDs	Gene.ontology..biological.process.
O00330	19,03765	19,82546	0	617;;sp|O00330|ODPX_HUMAN Pyruvate dehydrogenase prote	GO:0005739; (	mitochondrial acetyl-CoA biosynthetic process from pyruvate [GO:0061732]; pyruvate metabolic process [GO:0006090]
O00483	21,7762	22,73432	0	927;;sp|O00483|NDUA4_HUMAN Cytochrome c oxidase subuni	GO:0005739; (	mitochondrial electron transport, cytochrome c to oxygen [GO:0006123]; mitochondrial electron transport, NADH to ubiquinon [GO:0006120]; positive regulation of cytochrome-c oxidase activity [GO:1904960]; proton transmembrane transport
O14949	18,40399	20,08187	0	944;;sp|O14949|QCR8_HUMAN Cytochrome b-c1 complex subu	GO:0005739; (	cerebellar Purkinje cell layer development [GO:0021680]; hippocampus development [GO:0021766]; hypothalamus development [GO:0021854]; midbrain development [GO:0030901]; mitochondrial electron transport, ubiquinol to cytochrome (
O43678	17,24859	18,02923	0	982;;sp|O43678|NDUA2_HUMAN NADH dehydrogenase [ubiqu	GO:0001835; (	blastocyst hatching [GO:0001835]; mitochondrial electron transport, NADH to ubiquinone [GO:0006120]; mitochondrial respiratory chain complex I assembly [GO:0032981]
O60488	20,61414	21,53265	0	1003;;sp|O60488|ACSL4_HUMAN Long-chain-fatty-acid--CoA lig	GO:0001676; (	dendritic spine development [GO:0060996]; embryonic process involved in female pregnancy [GO:0060136]; fatty acid metabol process [GO:0006631]; fatty acid transport [GO:0015908]; lipid biosynthetic process [GO:0008610]; lipid metabolic process
O60783	20,97057	21,91911	0	1009;;sp|O60783|RT14_HUMAN 28S ribosomal protein S14, mit	GO:0003723; (	mitochondrial translation [GO:0032543]; mitochondrial translational elongation [GO:0070125]; mitochondrial translational termination [GO:0070126]; translation [GO:0006412]
O75306	19,39326	20,82858	0	1021;;sp|O75306|NDUS2_HUMAN NADH dehydrogenase [ubiqu	GO:0005654; (	cellular response to oxygen levels [GO:0071453]; gliogenesis [GO:0042063]; mitochondrial ATP synthesis coupled electron transport [GO:0042775]; mitochondrial electron transport, NADH to ubiquinone [GO:0006120]; mitochondrial respiratory chain
O75323	21,73583	23,11533	0	1022;;sp|O75323|NIPS2_HUMAN Protein NipSnap homolog 2 O	GO:0005739; (	mitochondrion organization [GO:0007005]; oxidative phosphorylation [GO:0006119]; positive regulation of high voltage-gated calcium channel activity [GO:1901843]
O75390	18,71535	20,64825	0	150;;sp|O75390|CISY_HUMAN Citrate synthase, mitochondrial (	GO:0003723; (	carbohydrate metabolic process [GO:0005975]; citrate metabolic process [GO:0006101]; tricarboxylic acid cycle [GO:0006099]
O75879	19,39891	20,37273	0	508;;sp|O75879|GATB_HUMAN Glutamyl-tRNA(Gln) amidotrans	GO:0005524; (	glutaminyl-tRNAGln biosynthesis via transamidation [GO:0070681]; mitochondrial translation [GO:0032543]
O96011	19,63341	20,56421	0	1093;;sp|O96011|PX11B_HUMAN Peroxisomal membrane prot	GO:0005654; (	peroxisome fission [GO:0016559]; peroxisome organization [GO:0007031]; protein import into peroxisome membrane [GO:0045046]; regulation of peroxisome size [GO:0044375]; signal transduction [GO:0007165]
P05166	18,92599	19,29766	0	441;;sp|P05166|PCCB_HUMAN Propionyl-CoA carboxylase beta	GO:0004658; (	biotin metabolic process [GO:0006768]; short-chain fatty acid catabolic process [GO:0019626]
P06493	21,47689	21,79408	0	3;;sp|P06493|CDK1_HUMAN Cyclin-dependent kinase 1 OS=Ho	GO:0000086; (	anaphase-promoting complex-dependent catabolic process [GO:0031145]; apoptotic process [GO:0006915]; cell division [GO:0051301]; cell migration [GO:0016477]; centrosome cycle [GO:0007098]; ciliary basal body-plasma membrane docking
P16219	19,35645	20,12591	0	579;;sp|P16219|ACADS_HUMAN Short-chain specific acyl-CoA c	GO:0003995; (	butyrate catabolic process [GO:0046359]; fatty acid beta-oxidation [GO:0006635]; fatty acid beta-oxidation using acyl-CoA dehydrogenase [GO:0033539]
P20674	20,77868	21,52191	0	785;;sp|P20674|COX5A_HUMAN Cytochrome c oxidase subunit	GO:0004129; (	mitochondrial electron transport, cytochrome c to oxygen [GO:0006123]
P21912	17,91975	19,67644	0	1250;;sp|P21912|SDHB_HUMAN Succinate dehydrogenase [ubi	GO:0005654; (	aerobic respiration [GO:0009060]; respiratory electron transport chain [GO:0022904]; succinate metabolic process [GO:0006105]; tricarboxylic acid cycle [GO:0006099]
P28288	19,62214	20,18483	0	1290;;sp|P28288|ABCD3_HUMAN ATP-binding cassette sub-fan	GO:0005324; (	fatty acid beta-oxidation [GO:0006635]; fatty acid biosynthetic process [GO:0006633]; lipid transport [GO:0006869]; long-chain fatty acid import into peroxisome [GO:0015910]; peroxisome organization [GO:0007031]; protein import into peroxisome
P30049	25,30921	26,03727	0	1298;;sp|P30049|ATPD_HUMAN ATP synthase subunit delta, m	GO:0000275; (	aerobic respiration [GO:0009060]; ATP biosynthetic process [GO:0006754]; ATP synthesis coupled proton transport [GO:0015986]; cristae formation [GO:0042407]; mitochondrial ATP synthesis coupled proton transport [GO:0042776];
P31947	24,18154	24,86074	0	1317;;sp|P31947|1433S_HUMAN 14-3-3 protein sigma OS=Hom	GO:0000079; (	DNA damage response, signal transduction by p53 class mediator resulting in cell cycle arrest [GO:0006977]; establishment of skin barrier [GO:0061436]; intrinsic apoptotic signaling pathway in response to DNA damage [GO:0008630]; keratinization
P47897	19,68637	19,89207	0	188;;sp|P47897|SYQ_HUMAN Glutamine--tRNA ligase OS=Hom	GO:0004819; (	brain development [GO:0007420]; glutaminyl-tRNA aminoacylation [GO:0006425]; negative regulation of apoptotic signaling pathway [GO:2001234]; negative regulation of protein kinase activity [GO:0006469]; negative regulation of stress-activated
P49590	19,28039	20,06855	0	225;;sp|P49590|SYHM_HUMAN Probable histidine--tRNA ligase	GO:0003723; (	histidyl-tRNA aminoacylation [GO:0006427]; translation [GO:0006412]; tRNA aminoacylation for protein translation [GO:0006418]
P53597	20,17925	21,392	0	296;;sp|P53597|SUCA_HUMAN Succinate--CoA ligase [ADP/GDF	GO:0000166; (	tricarboxylic acid cycle [GO:0006099]
P56181	18,33974	20,50057	0	1478;;sp|P56181-2|NDUV3_HUMAN Isoform 2 of NADH dehydr	GO:0003723; (	mitochondrial ATP synthesis coupled electron transport [GO:0042775]; mitochondrial electron transport, NADH to ubiquinone [GO:0006120]; mitochondrial respiratory chain complex I assembly [GO:0032981]
P56556	20,22623	21,0071	0	151;;sp|P56556|NDUA6_HUMAN NADH dehydrogenase [ubiqui	GO:0005743; (	mitochondrial electron transport, NADH to ubiquinone [GO:0006120]; mitochondrial respiratory chain complex I assembly [GO:0032981]; response to oxidative stress [GO:0006979]
P61604	20,40052	21,06368	0	394;;sp|P61604|CH10_HUMAN 10 kDa heat shock protein, mito	GO:0001649; (	activation of cysteine-type endopeptidase activity involved in apoptotic process [GO:0006919]; chaperone cofactor-dependent protein refolding [GO:0051085]; osteoblast differentiation [GO:0001649]; protein folding [GO:0006457]; response to unfolded
P82914	19,37554	20,61944	0	1591;;sp|P82914|RT15_HUMAN 28S ribosomal protein S15, mit	GO:0003723; (	mitochondrial translation [GO:0032543]; mitochondrial translational elongation [GO:0070125]; mitochondrial translational termination [GO:0070126]; translation [GO:0006412]
Q12906	19,35055	19,26936	0	1659;;sp|Q12906|ILF3_HUMAN Interleukin enhancer-binding fa	GO:0003677; (	defense response to virus [GO:0051607]; negative regulation of transcription, DNA-templated [GO:0045892]; negative regulatic of translation [GO:0017148]; negative regulation of viral genome replication [GO:0045071]; positive regulation of transcription,
Q12981	19,82682	20,70969	0	1663;;sp|Q12981|SEC20_HUMAN Vesicle transport protein SEC	GO:0005484; (	apoptotic process [GO:0006915]; endoplasmic reticulum membrane fusion [GO:0016320]; endoplasmic reticulum organization [GO:0007029]; execution phase of apoptosis [GO:0097194]; negative regulation of apoptotic process [GO:0043066]; response t
Q13131	19,32424	19,10718	0	1670;;sp|Q13131|AAPK1_HUMAN 5-AMP-activated protein kin	GO:0000187; (	activation of MAPK activity [GO:0000187]; bile acid and bile salt transport [GO:0015721]; bile acid signaling pathway [GO:0038183]; CAMKK-AMPK signaling cascade [GO:0061762]; cell cycle arrest [GO:0007050]; cellular response to calcium ion
Q13501	21,27821	22,34643	0	535;;sp|Q13501|SQSTM_HUMAN Sequestosome-1 OS=Homo s	GO:0000122; (	aggrephagy [GO:0035973]; apoptotic process [GO:0006915]; autophagy [GO:0006914]; autophagy of mitochondrion [GO:0000422]; cell differentiation [GO:0030154]; endosomal transport [GO:0016197]; endosome organization [GO:0007032];
Q15388	22,58323	23,01071	0	1760;;sp|Q15388|TOM20_HUMAN Mitochondrial import recep	GO:0005739; (	macroautophagy [GO:0016236]; protein deubiquitination [GO:0016579]; protein import into mitochondrial matrix [GO:0030150]; protein targeting to mitochondrion [GO:0006626]; response to 3,3',5-triiodo-L-thyronine [GO:1905242]; respons
Q16543	21,61248	21,79765	0	1778;;sp|Q16543|CDC37_HUMAN Hsp90 co-chaperone Cdc37 (	GO:0000079; (	ERBB2 signaling pathway [GO:0038128]; positive regulation of mitophagy in response to mitochondrial depolarization [GO:0098779]; posttranscriptional regulation of gene expression [GO:0010608]; protein folding [GO:0006457]; protein
Q16698	19,41932	20,96663	0	517;;sp|Q16698|DECR_HUMAN 2,4-dienoyl-CoA reductase, mit	GO:0005634; (	fatty acid beta-oxidation [GO:0006635]; positive regulation of cold-induced thermogenesis [GO:0120162]
Q3SXM5	20,1566	21,67848	0	1790;;sp|Q3SXM5|HSDL1_HUMAN Inactive hydroxysteroid deh	GO:0005739;	GO:0016491; GO:0043231; GO:0045111
Q5HYK3	19,82198	21,06098	0	663;;sp|Q5HYK3|COQ5_HUMAN 2-methoxy-6-polyprenyl-1,4-b	GO:0005759; (	methylation [GO:0032259]; ubiquinone biosynthetic process [GO:0006744]
Q5SRD1	20,32784	21,58587	0	943;;sp|Q5SRD1|TI23B_HUMAN Putative mitochondrial import	GO:0005744; (	protein import into mitochondrial matrix [GO:0030150]
Q6P161	19,93905	21,20807	0	1863;;sp|Q6P161|RM54_HUMAN 39S ribosomal protein L54, m	GO:0003723; (	mitochondrial translational elongation [GO:0070125]; mitochondrial translational termination [GO:0070126]
Q6YN16	20,14034	20,53098	0	1882;;sp|Q6YN16|HSDL2_HUMAN Hydroxysteroid dehydrogen	GO:0005739;	GO:0005777; GO:0016020; GO:0016491
Q8N357	21,14271	22,07865	0	1955;;sp|Q8N357|S35F6_HUMAN Solute carrier family 35 mem	GO:0005654; (	negative regulation of mitochondrial outer membrane permeabilization involved in apoptotic signaling pathway [GO:1901029]; positive regulation of cell population proliferation [GO:0008284]
Q8N4Q1	23,58566	24,18586	0	1958;;sp|Q8N4Q1|MIA40_HUMAN Mitochondrial intermembra	GO:0005739; (	'de novo' posttranslational protein folding [GO:0051084]; mitochondrial respiratory chain complex assembly [GO:0033108]; peptidyl-cysteine oxidation [GO:0018171]; protein import into mitochondrial intermembrane space [GO:0045041]; protein
Q8N5G0	21,31213	22,19012	0	1962;;sp|Q8N5G0|SIM20_HUMAN Small integral membrane pr	GO:0005576; (	mitochondrial cytochrome c oxidase assembly [GO:0033617]
Q8NEN9	19,8634	20,43573	0	1985;;sp|Q8NEN9|PDZD8_HUMAN PDZ domain-containing prot	GO:0005739; (	cytoskeleton organization [GO:0007010]; intracellular signal transduction [GO:0035556]; lipid transport [GO:0006869];
Q8NI60	18,85111	21,29283	0	1993;;sp|Q8NI60|COQ8A_HUMAN Atypical kinase COQ8A, mito	GO:0005524; (	phosphorylation [GO:0016310]; protein phosphorylation [GO:0006468]; ubiquinone biosynthetic process [GO:0006744]
Q96A26	19,90345	21,77302	0	690;;sp|Q96A26|F162A_HUMAN Protein FAM162A OS=Homo s	GO:0005739; (	activation of cysteine-type endopeptidase activity involved in apoptotic process [GO:0006919]; cellular response to hypoxia [GO:0071456]; neuron apoptotic process [GO:0051402]; positive regulation of apoptotic process [GO:0043065]; positive
Q96EK5	20,55009	21,70582	0	203;;sp|Q96EK5|KBP_HUMAN KIF1-binding protein OS=Homo s	GO:0005739; (	cell differentiation [GO:0030154]; mitochondrial transport [GO:0006839]; nervous system development [GO:0007399]
Q96GC5	19,48048	20,17292	0	657;;sp|Q96GC5|RM48_HUMAN 39S ribosomal protein L48, mit	GO:0005739; (	mitochondrial translational elongation [GO:0070125]; mitochondrial translational termination [GO:0070126]
Q99417	19,52139	20,62587	0	37;;sp|Q99417|MYCBP_HUMAN c-Myc-binding protein OS=Hon	GO:0003713; (	regulation of transcription, DNA-templated [GO:0006355]; spermatogenesis [GO:0007283]
Q99807	20,27776	21,27696	0	786;;sp|Q99807|COQ7_HUMAN 5-demethoxyubiquinone hydro	GO:0000122; (	determination of adult lifespan [GO:0008340]; negative regulation of transcription by RNA polymerase II [GO:0000122]; positive regulation of transcription by RNA polymerase II [GO:0045944]; regulation of gene expression [GO:0010468]; regulation of
Q9BPW8	20,41421	21,20365	0	2147;;sp|Q9BPW8|NIPS1_HUMAN Protein NipSnap homolog 1 (	GO:0005739; (	sensory perception of pain [GO:0019233]
Q9BYD3	20,75222	22,09896	0	880;;sp|Q9BYD3|RM04_HUMAN 39S ribosomal protein L4, mitc	GO:0003723; (	mitochondrial translational elongation [GO:0070125]; mitochondrial translational termination [GO:0070126]
Q9H2V7	22,1456	22,10981	0	775;;sp|Q9H2V7|SPNS1_HUMAN Protein spinster homolog 1 O	GO:0005743; (	lipid transport [GO:0006869]
Q9P0M9	19,91716	21,38906	0	495;;sp|Q9P0M9|RM27_HUMAN 39S ribosomal protein L27, mi	GO:0003723; (	mitochondrial translational elongation [GO:0070125]; mitochondrial translational termination [GO:0070126]; translation
Q9Y277	19,63028	20,6856	0	2404;;sp|Q9Y277|VDAC3_HUMAN Voltage-dependent anion-se	GO:0000166; (	adenine transport [GO:0015853]; regulation of cilium assembly [GO:1902017]
Q9Y5Z9	19,75181	20,97106	0	2448;;sp|Q9Y5Z9|UBIA1_HUMAN UbiA prenyltransferase doma	GO:0004659; (	menaquinone biosynthetic process [GO:0009234]; ubiquinone biosynthetic process [GO:0006744]; ubiquinone biosynthetic process via 3,4-dihydroxy-5-polyprenylbenzoate [GO:0032194]; vitamin K biosynthetic process [GO:0042371]; vitamin K
